# Supplementary figures and images for: Functional membrane microdomains and the hydroxamate siderophore transporter ATPase FhuC govern Isd-dependent heme acquisition in Staphylococcus aureus
Source: eLife. 2023 Apr 12;12:e85304. doi: 10.7554/eLife.85304 (PMC10147376; doi:10.7554/eLife.85304)

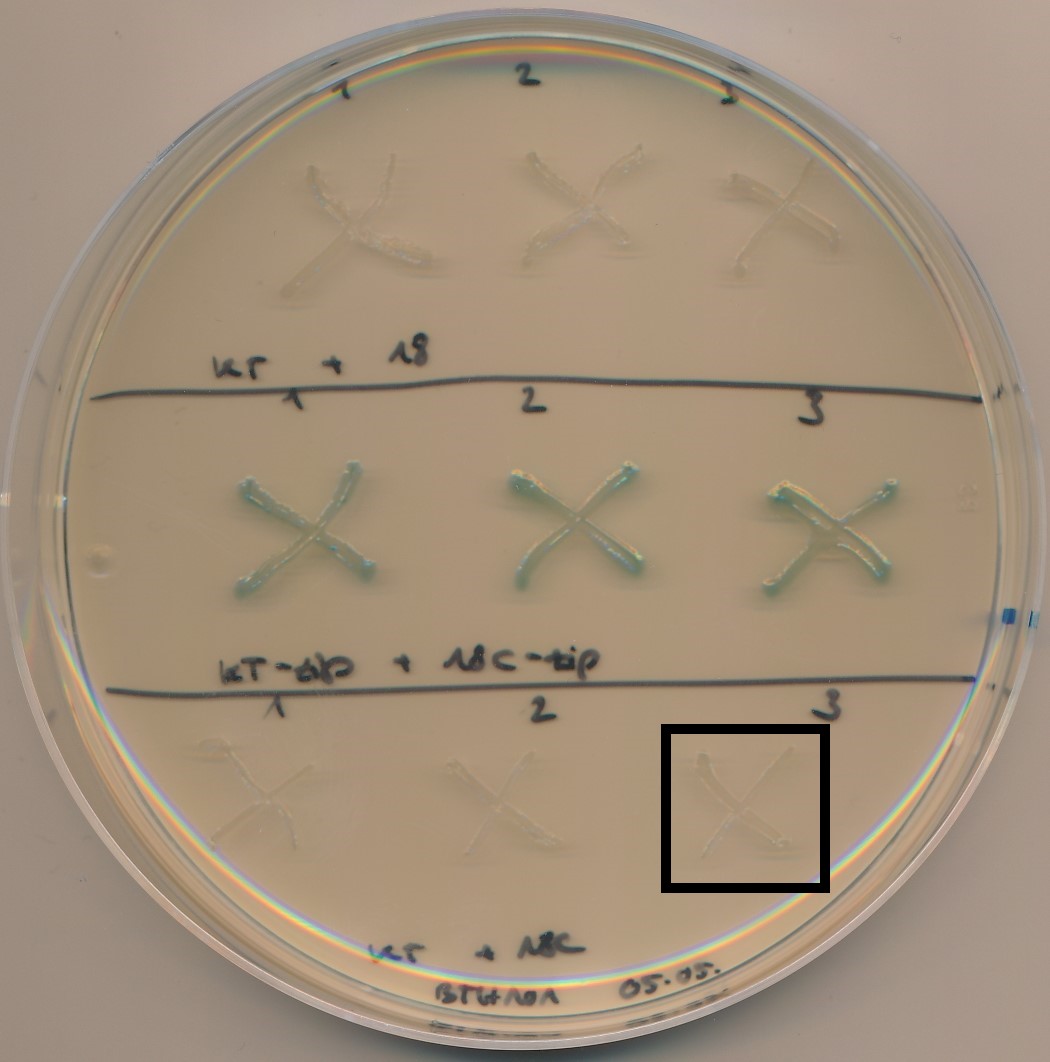

Supplement: Figure 2—source data 1. [file elife-85304-fig2-data1.zip › Figure 2a-source data/empty vector control_annotated.jpg]

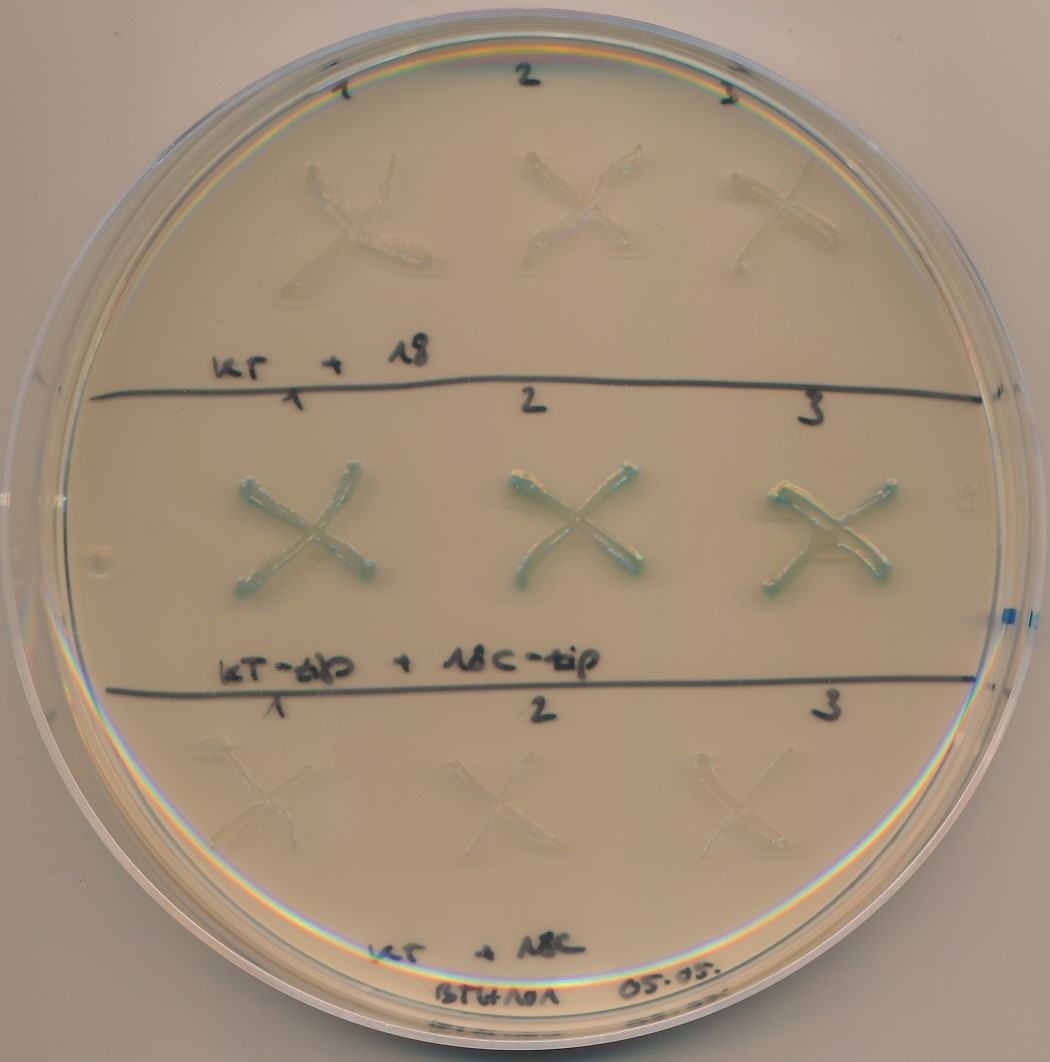

Supplement: Figure 2—source data 1. [file elife-85304-fig2-data1.zip › Figure 2a-source data/empty vector control_original.jpg]

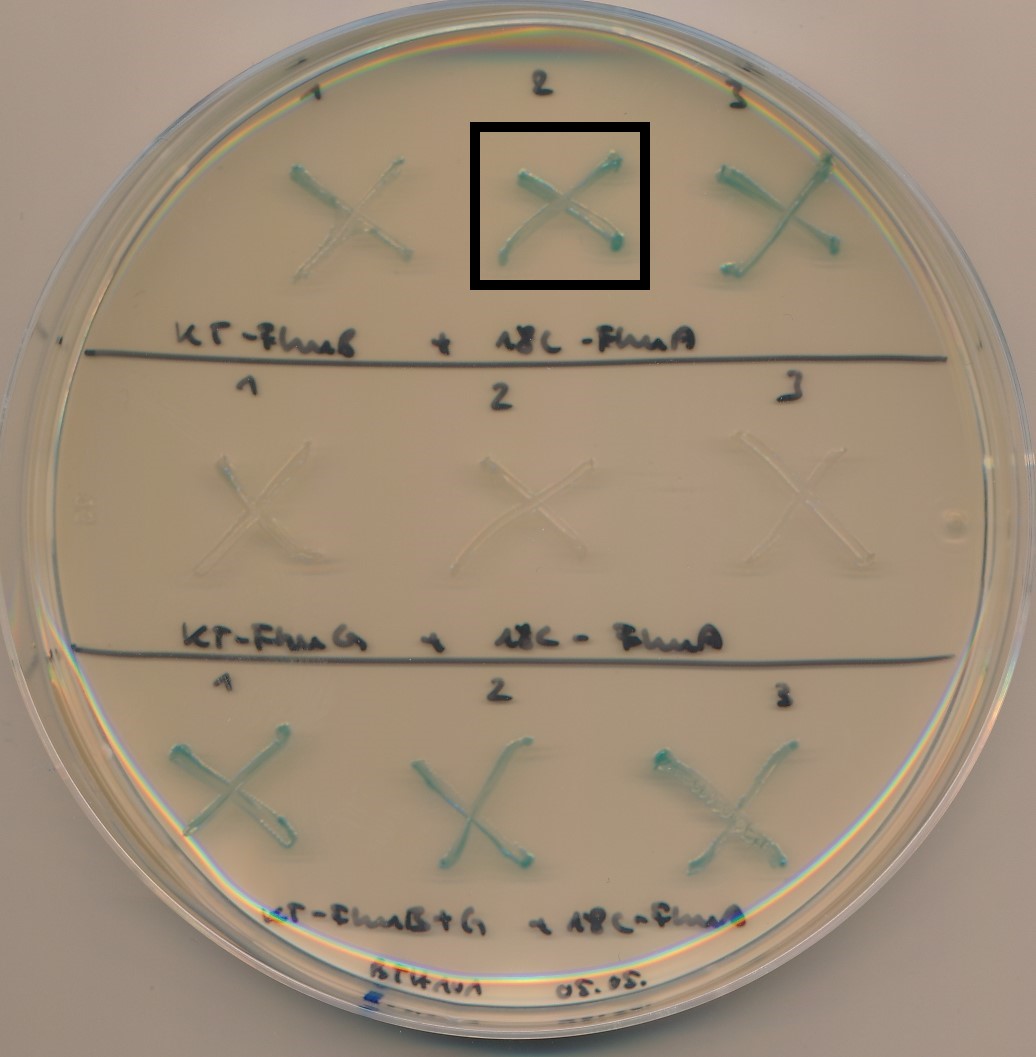

Supplement: Figure 2—source data 1. [file elife-85304-fig2-data1.zip › Figure 2a-source data/FhuB+FhuC_annotated.jpg]

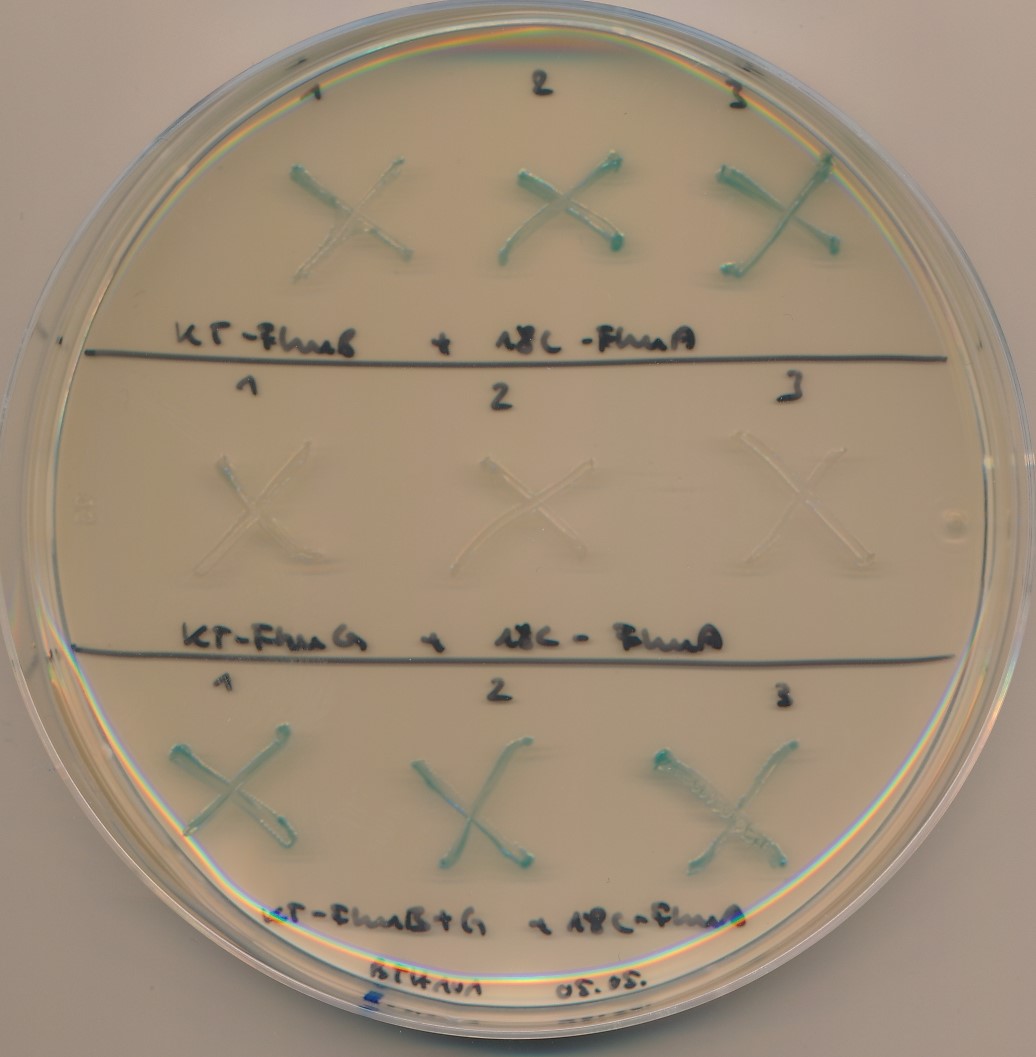

Supplement: Figure 2—source data 1. [file elife-85304-fig2-data1.zip › Figure 2a-source data/FhuB+FhuC_original.jpg]

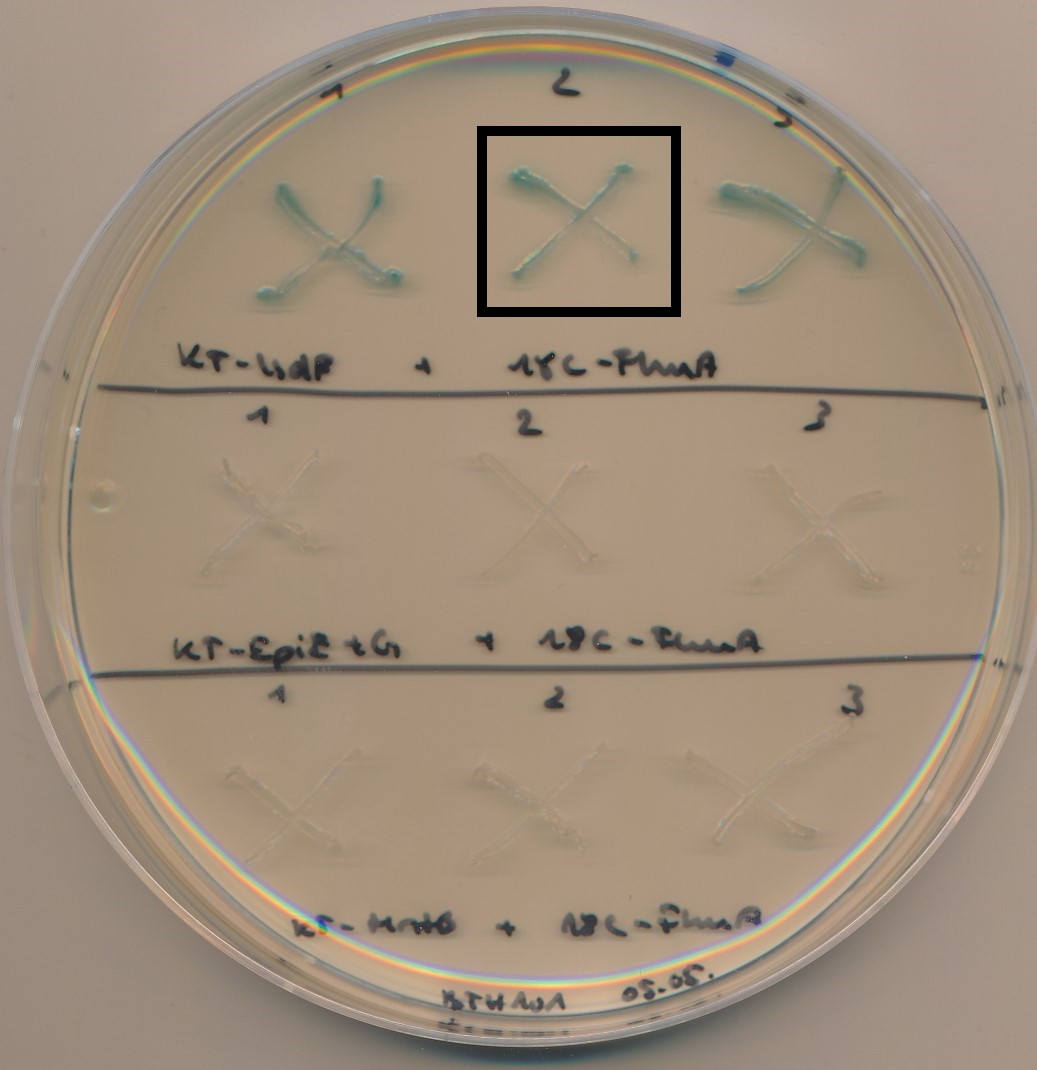

Supplement: Figure 2—source data 1. [file elife-85304-fig2-data1.zip › Figure 2a-source data/IsdF+FhuC_annotated.jpg]

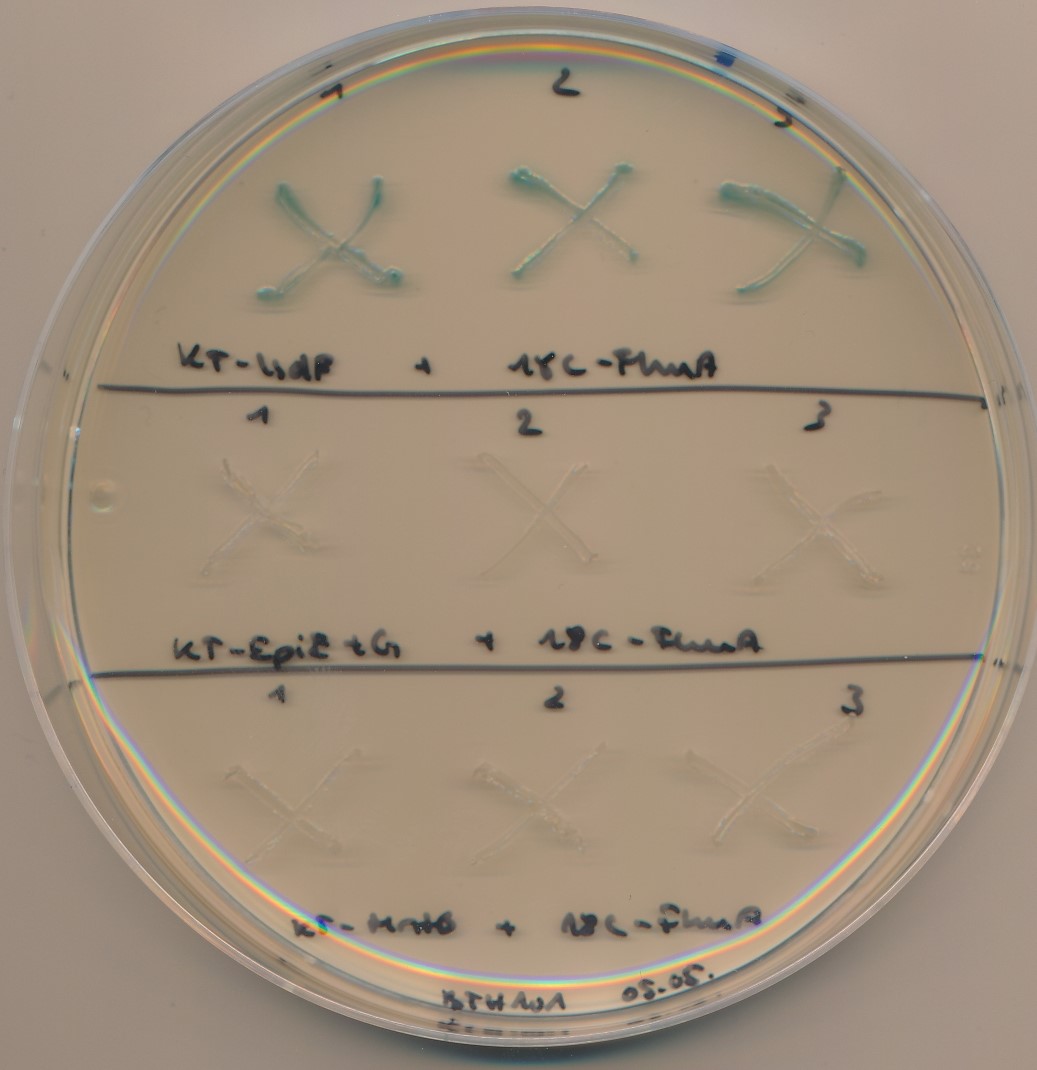

Supplement: Figure 2—source data 1. [file elife-85304-fig2-data1.zip › Figure 2a-source data/IsdF+FhuC_original.jpg]

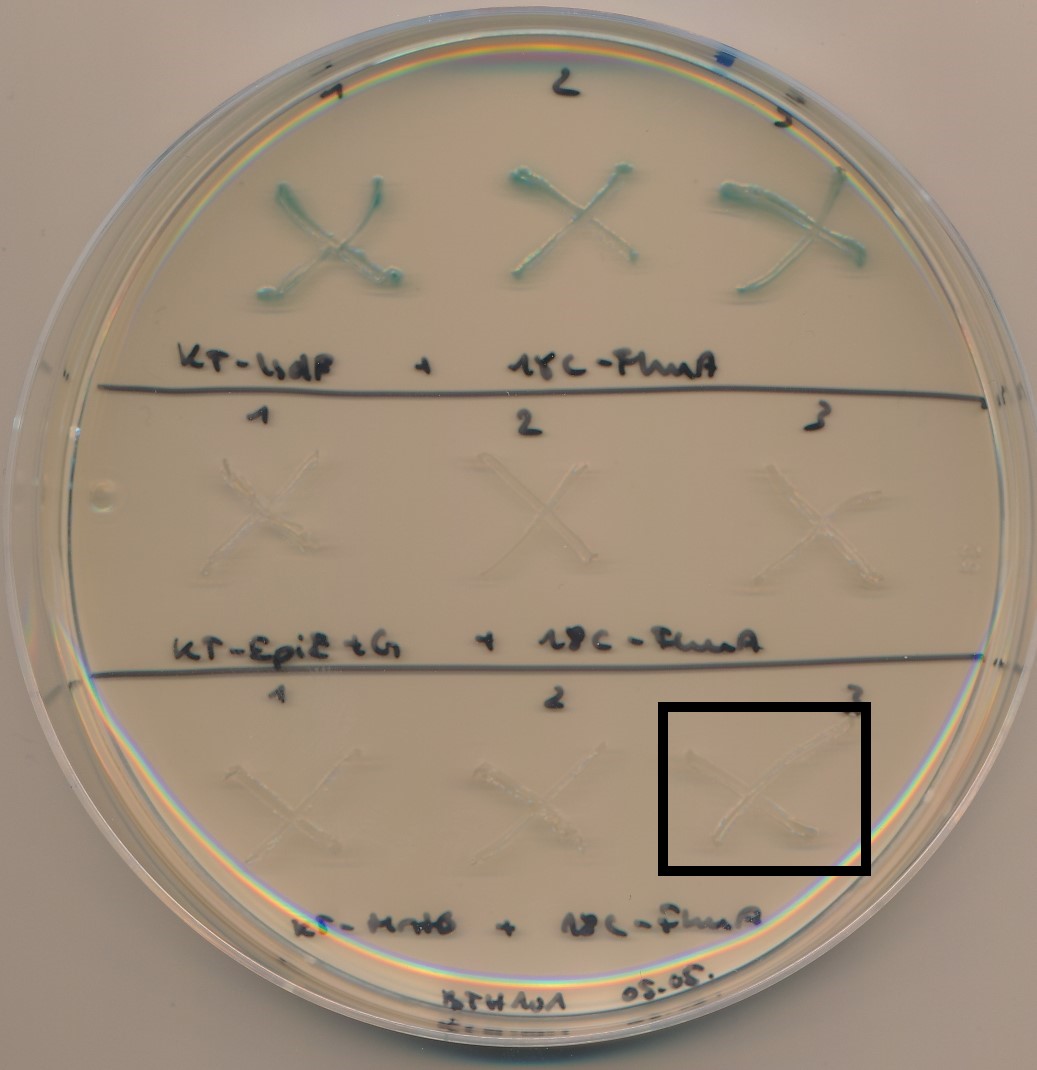

Supplement: Figure 2—source data 1. [file elife-85304-fig2-data1.zip › Figure 2a-source data/MntB+FhuC_annotated.jpg]

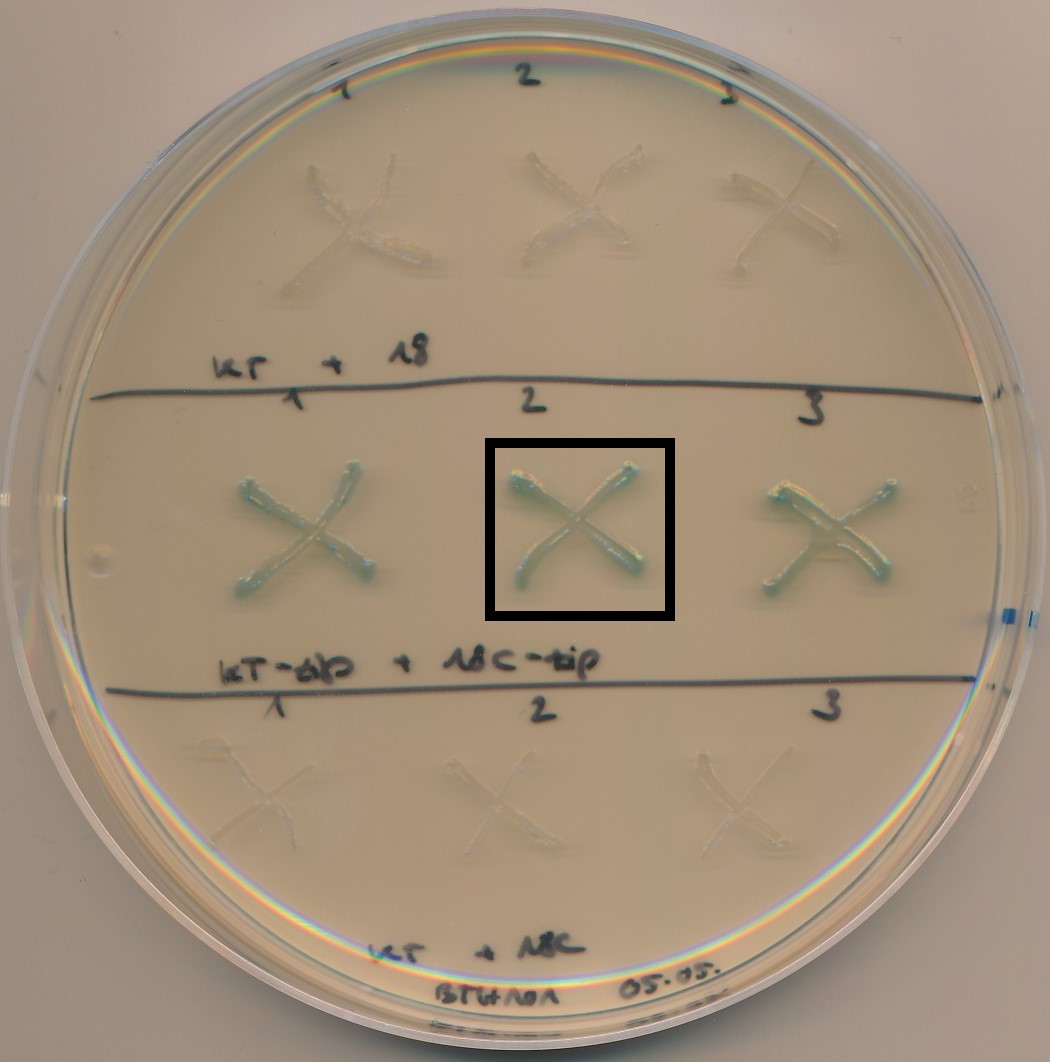

Supplement: Figure 2—source data 1. [file elife-85304-fig2-data1.zip › Figure 2a-source data/zip control_annotated.jpg]

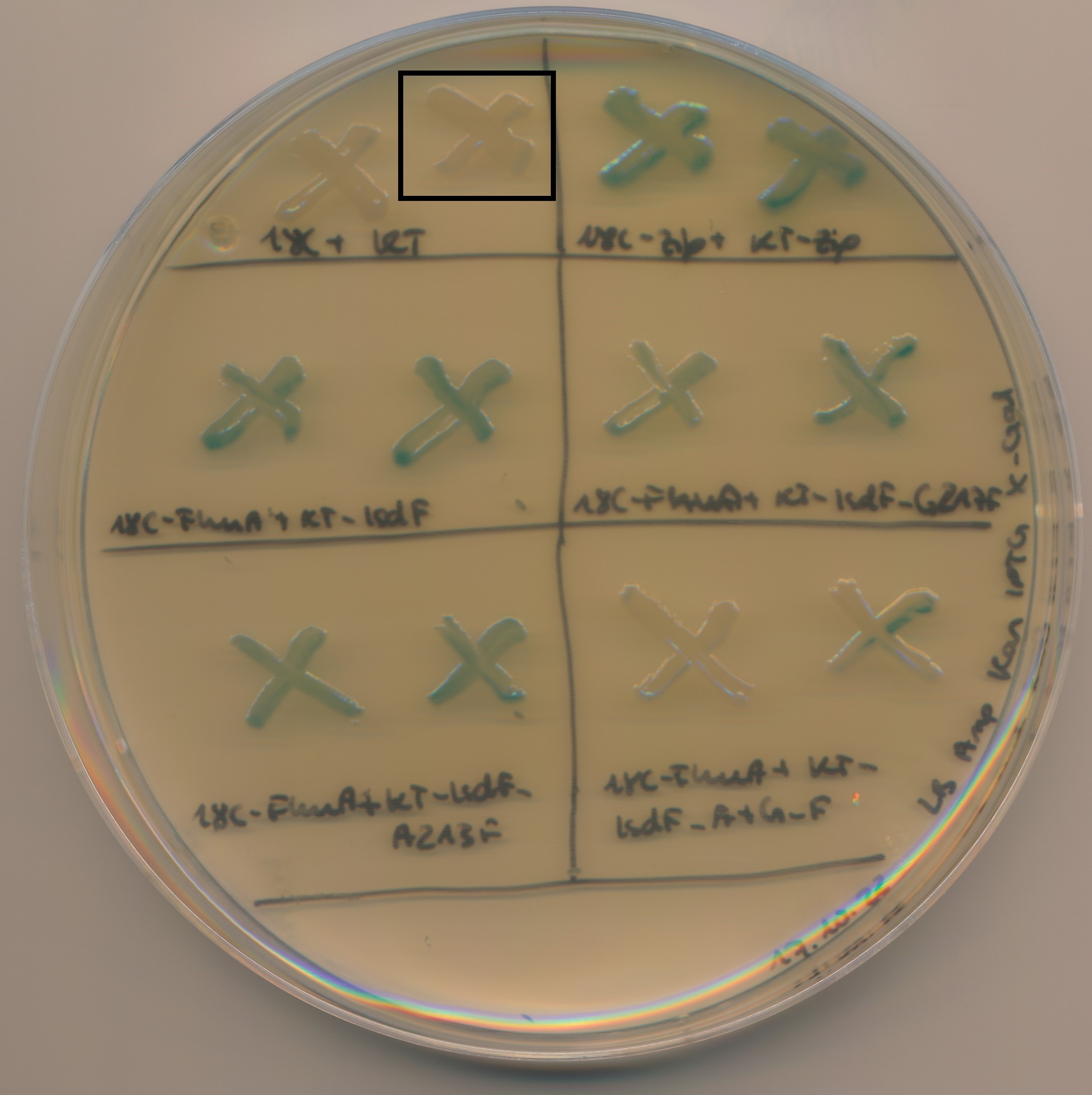

Supplement: Figure 2—source data 1. [file elife-85304-fig2-data1.zip › Figure 2d-source data/empty vector control_annotated.jpg]

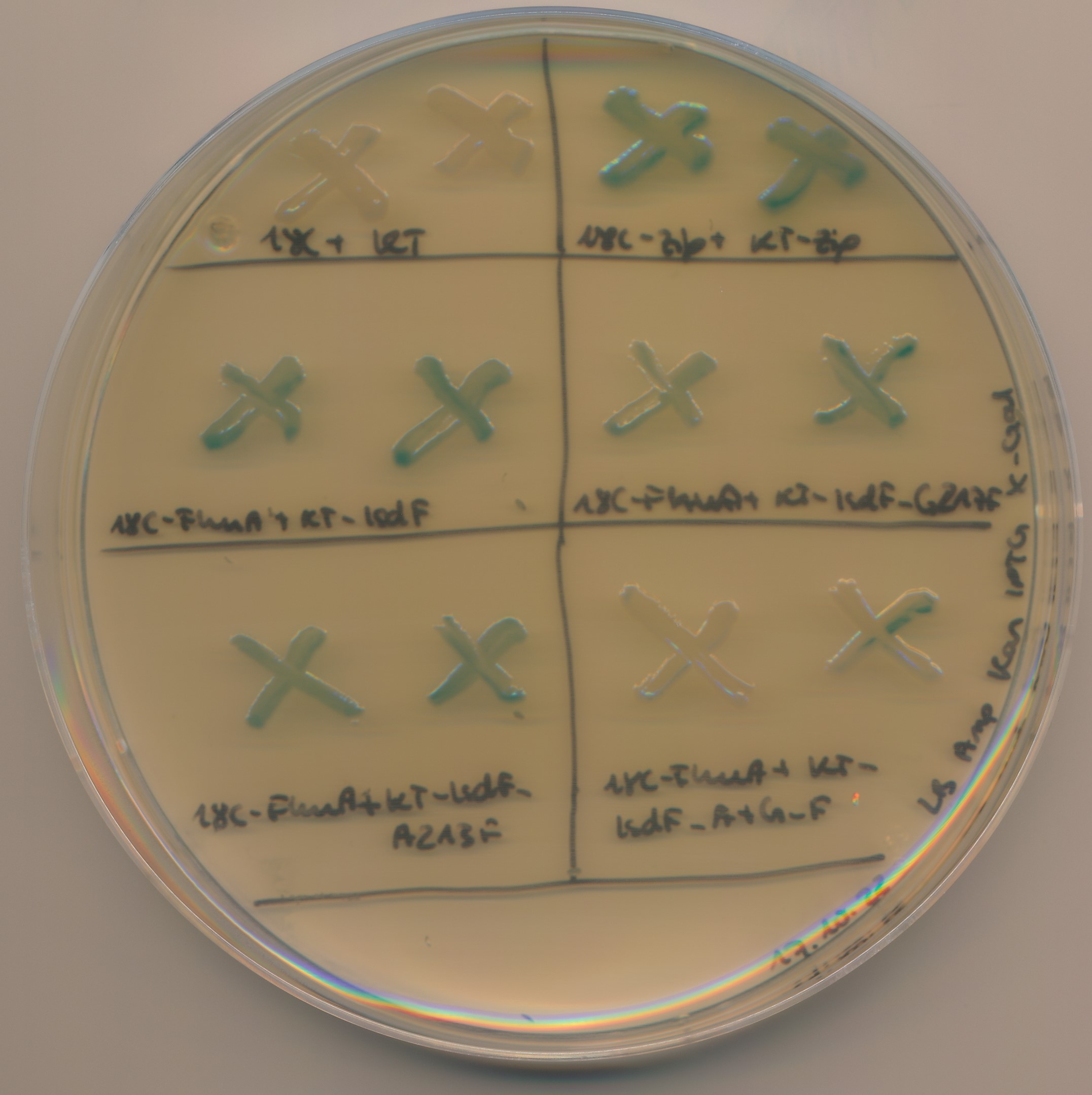

Supplement: Figure 2—source data 1. [file elife-85304-fig2-data1.zip › Figure 2d-source data/empty vector control_original.jpg]

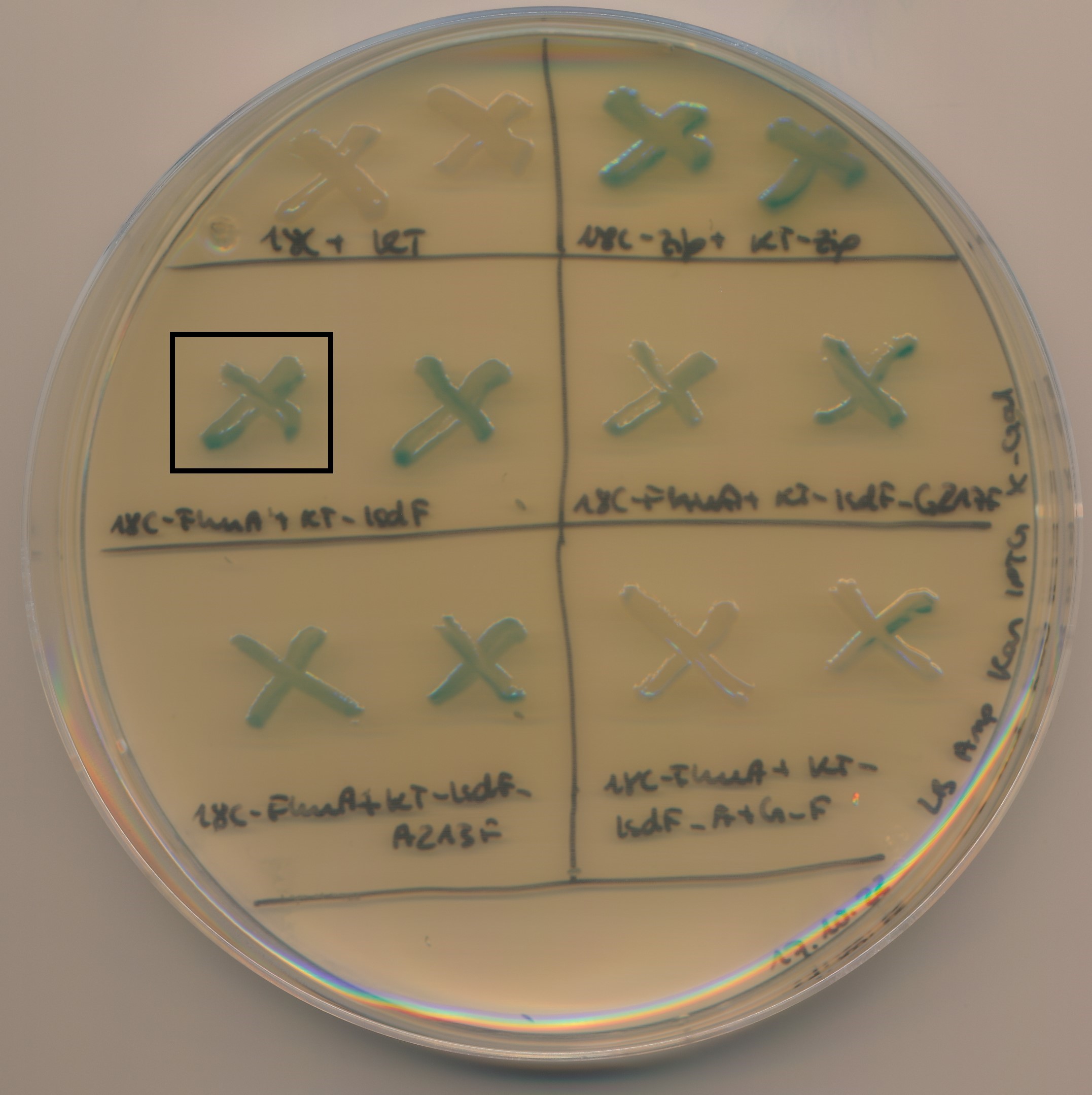

Supplement: Figure 2—source data 1. [file elife-85304-fig2-data1.zip › Figure 2d-source data/IsdF+FhuC_annotated.jpg]

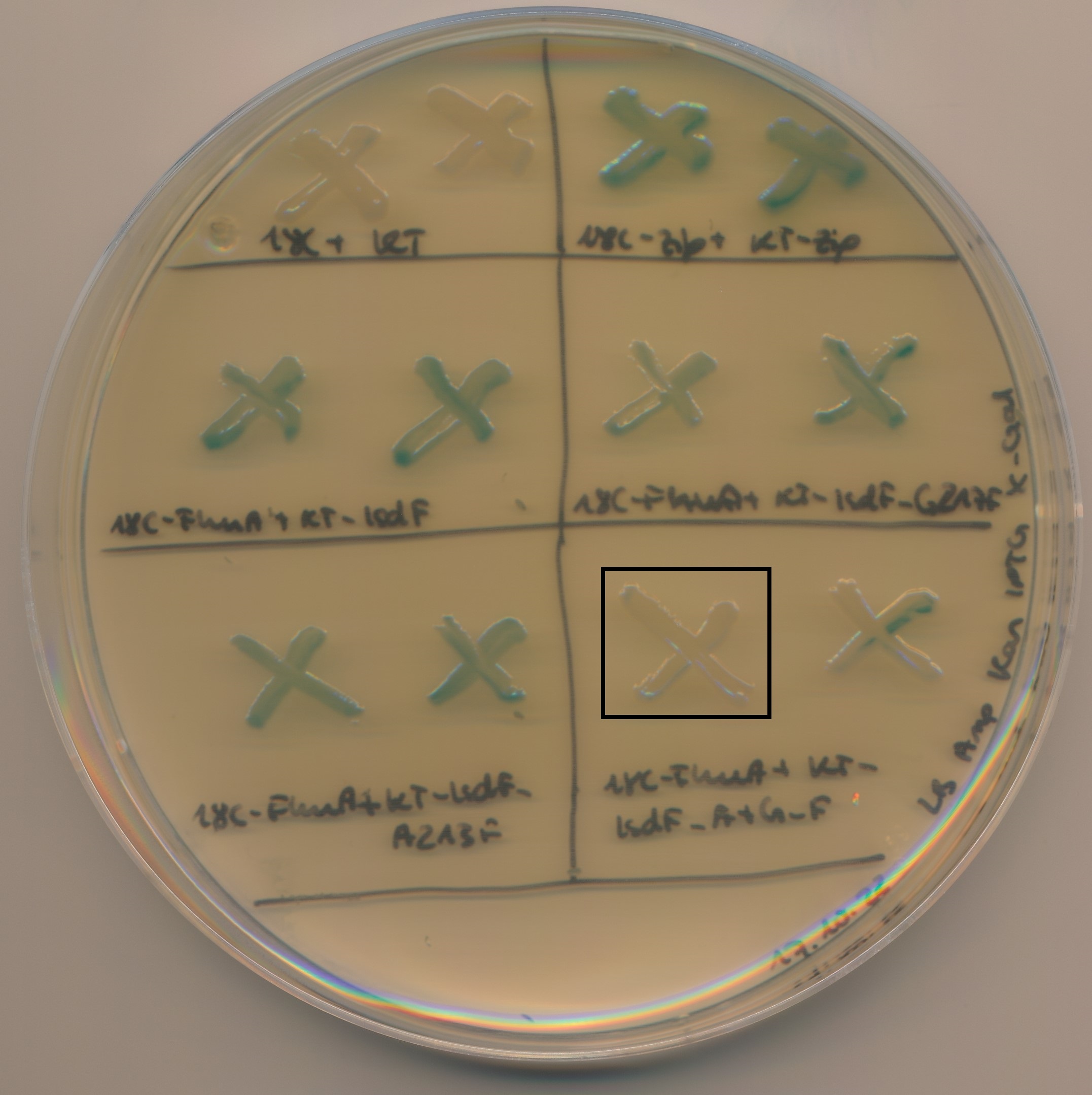

Supplement: Figure 2—source data 1. [file elife-85304-fig2-data1.zip › Figure 2d-source data/IsdFA+G_F+FhuC_annotated.jpg]

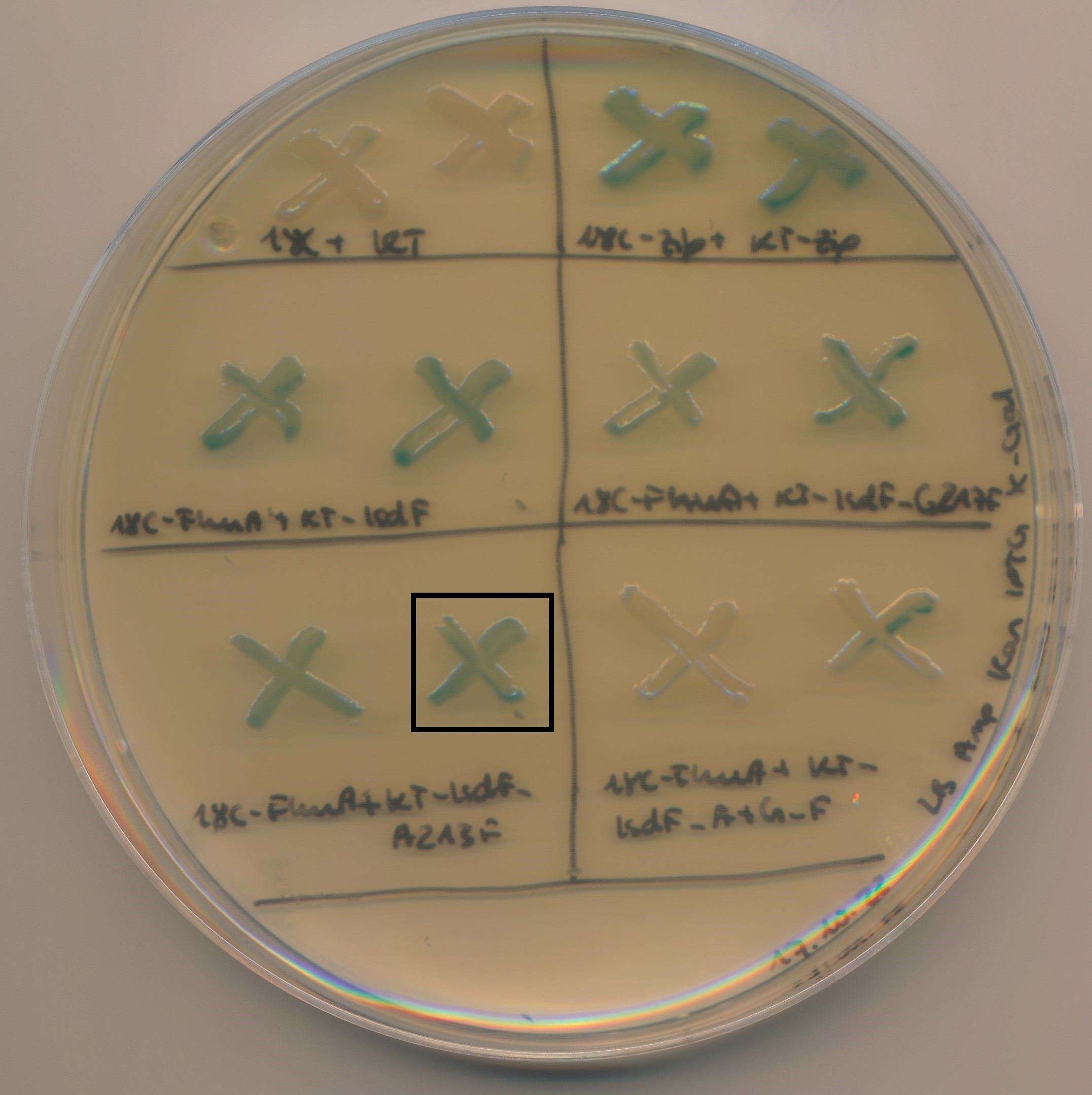

Supplement: Figure 2—source data 1. [file elife-85304-fig2-data1.zip › Figure 2d-source data/IsdFA213F+FhuC_annotated.jpg]

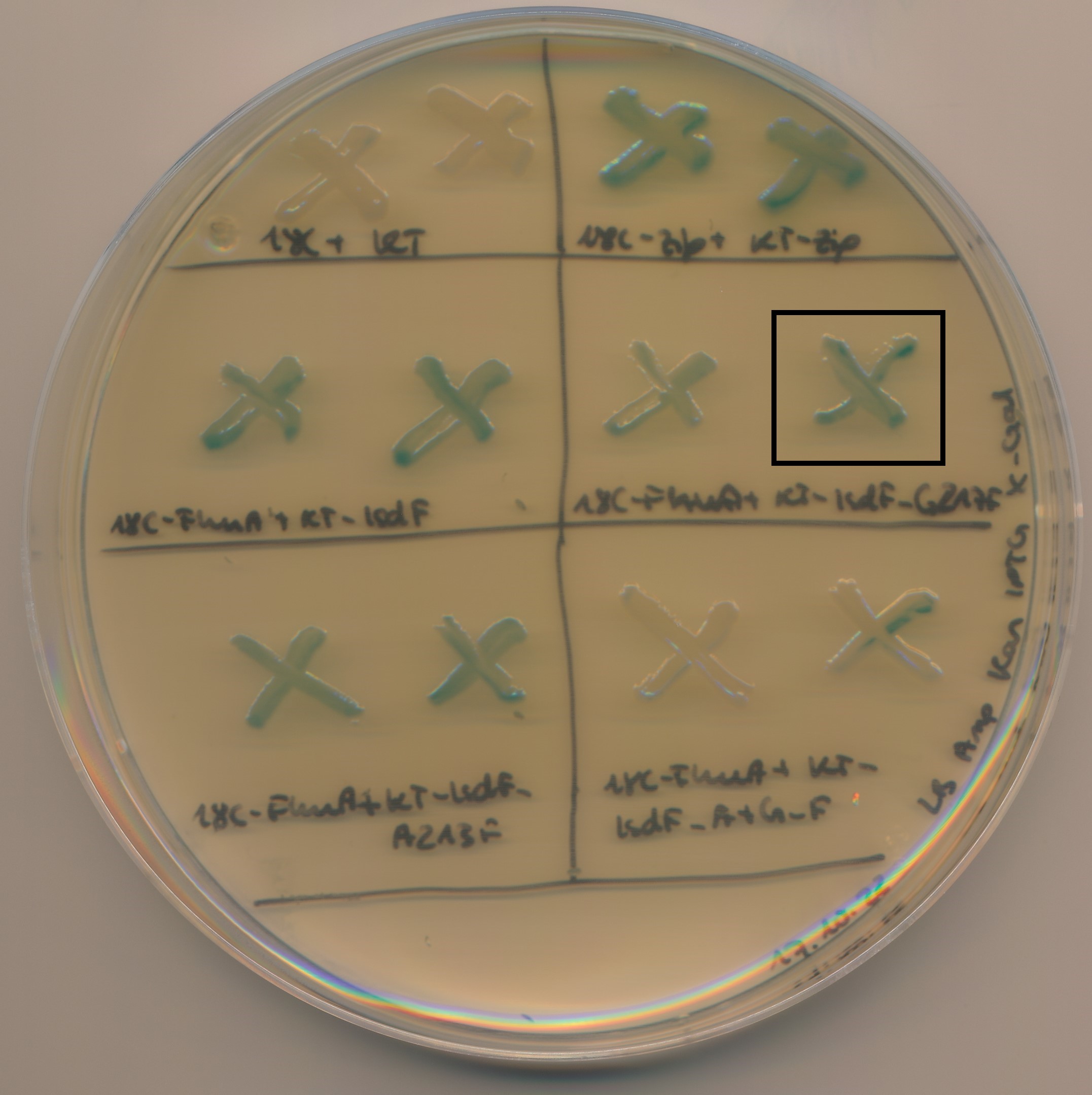

Supplement: Figure 2—source data 1. [file elife-85304-fig2-data1.zip › Figure 2d-source data/IsdFG217F+FhuC_annotated.jpg]

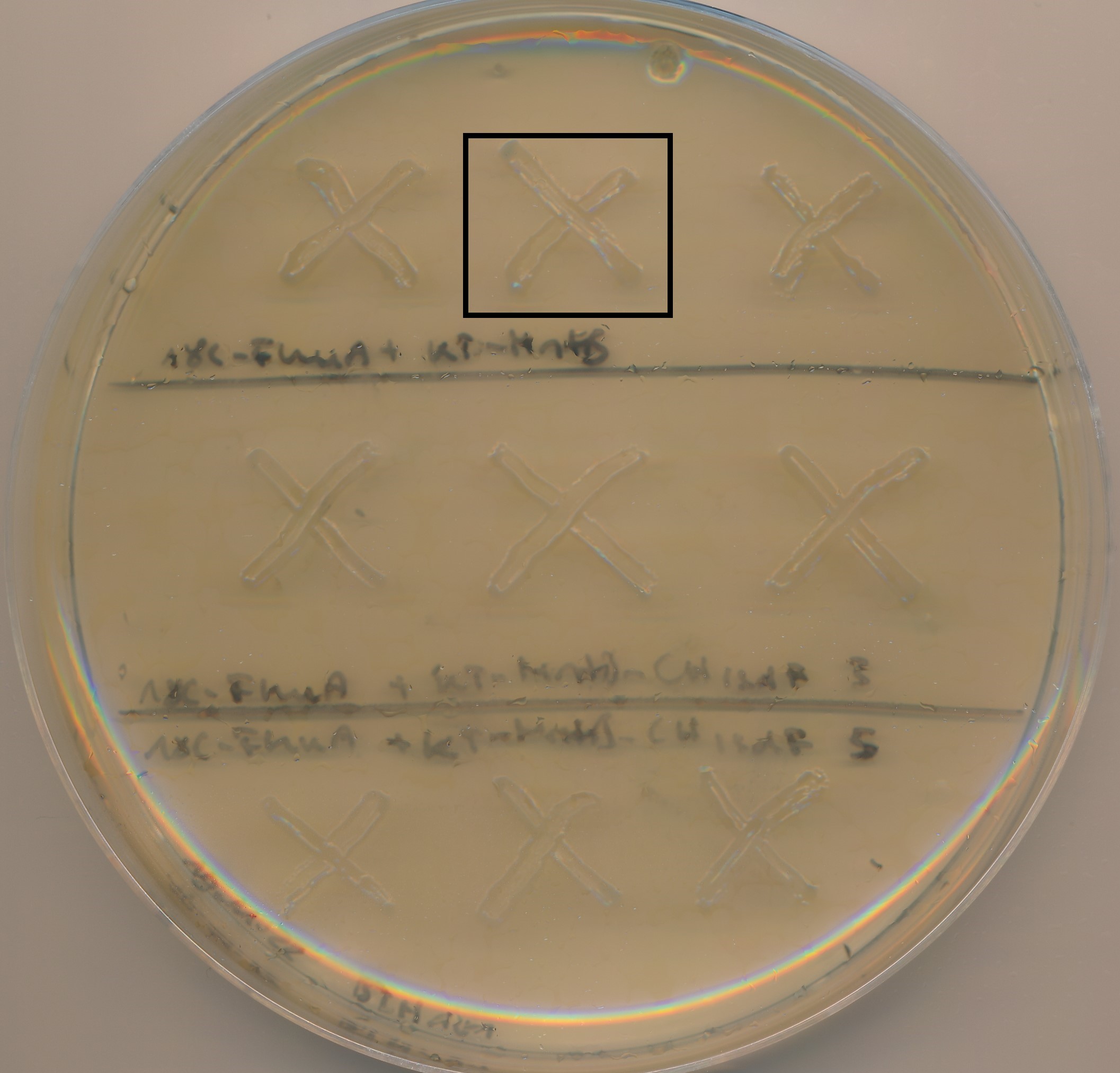

Supplement: Figure 2—source data 1. [file elife-85304-fig2-data1.zip › Figure 2d-source data/MntB+FhuC_annotated.jpg]

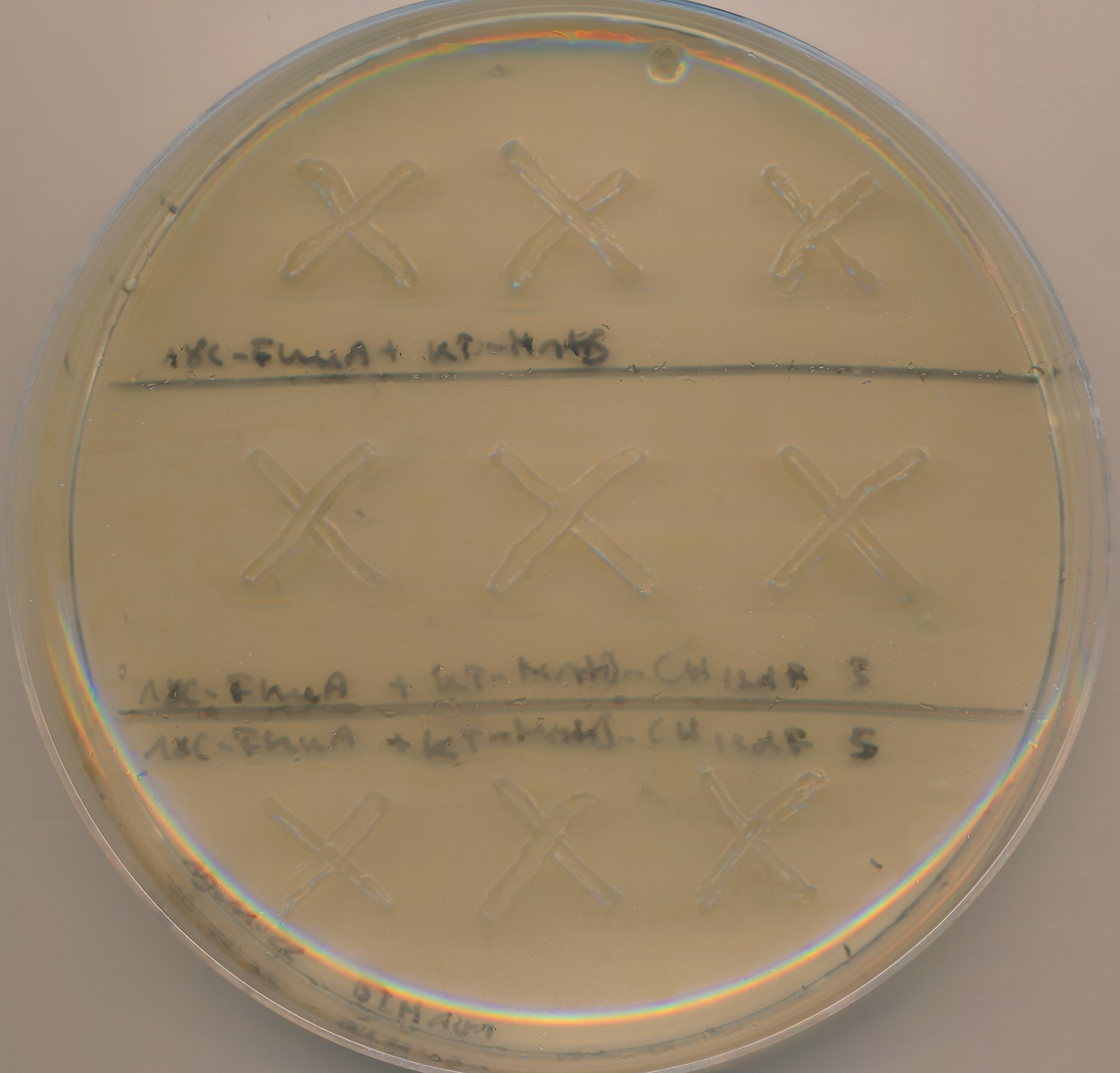

Supplement: Figure 2—source data 1. [file elife-85304-fig2-data1.zip › Figure 2d-source data/MntB+FhuC_original.jpg]

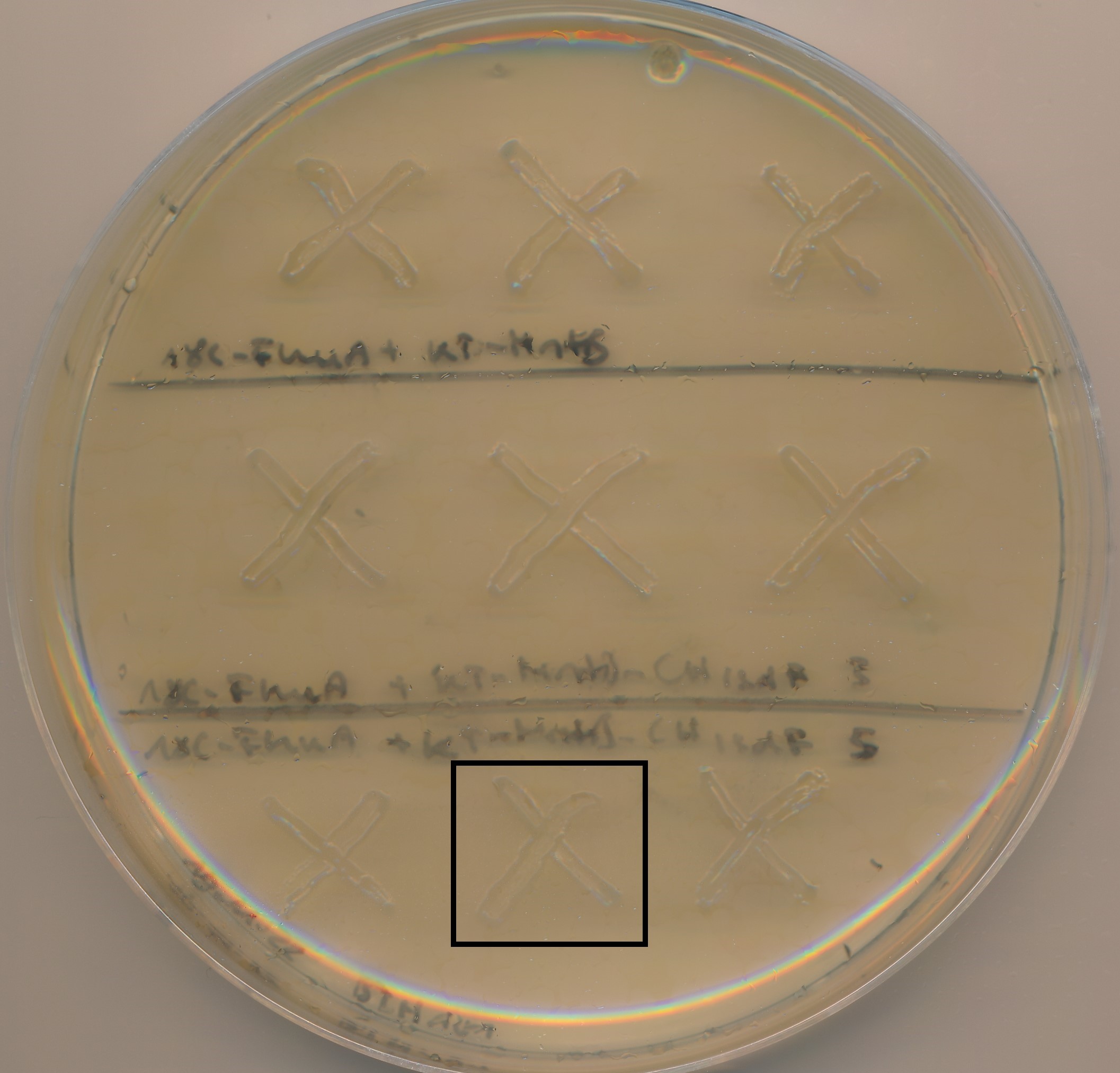

Supplement: Figure 2—source data 1. [file elife-85304-fig2-data1.zip › Figure 2d-source data/MntB-CHisdF+FhuC_annotated.jpg]

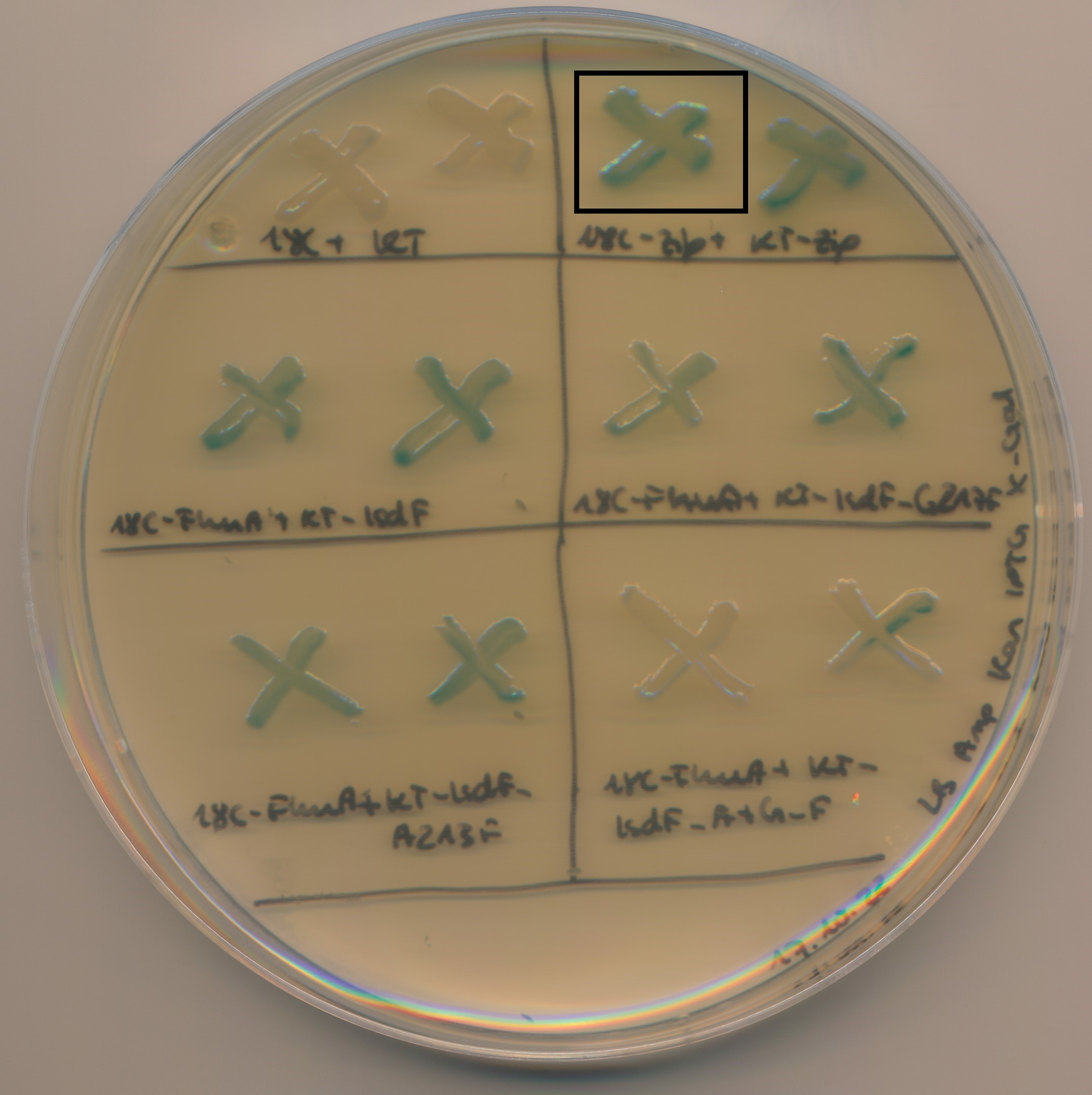

Supplement: Figure 2—source data 1. [file elife-85304-fig2-data1.zip › Figure 2d-source data/zip control_annotated.jpg]

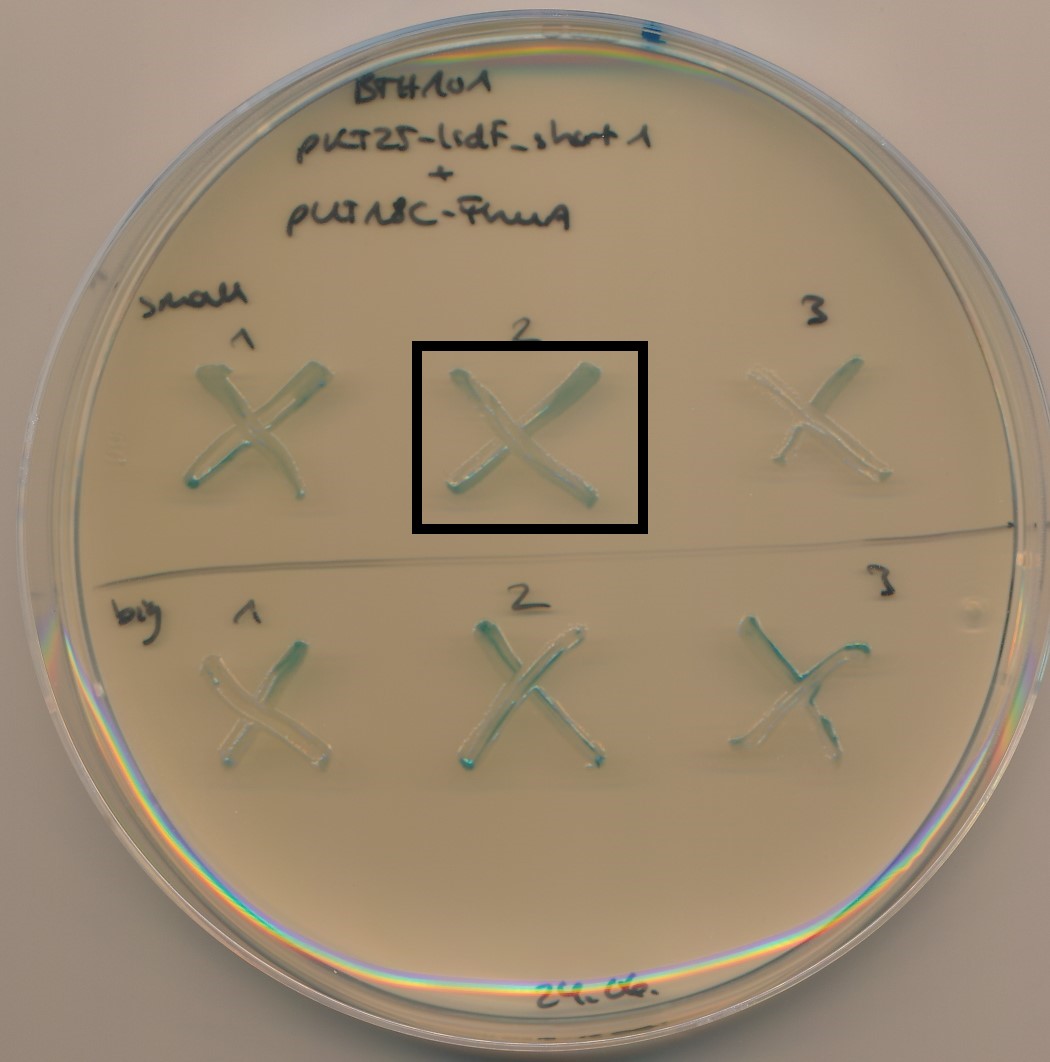

Supplement: Figure 2—source data 1. [file elife-85304-fig2-data1.zip › Figure 2e-source data/short1+FhuC_annotated.jpg]

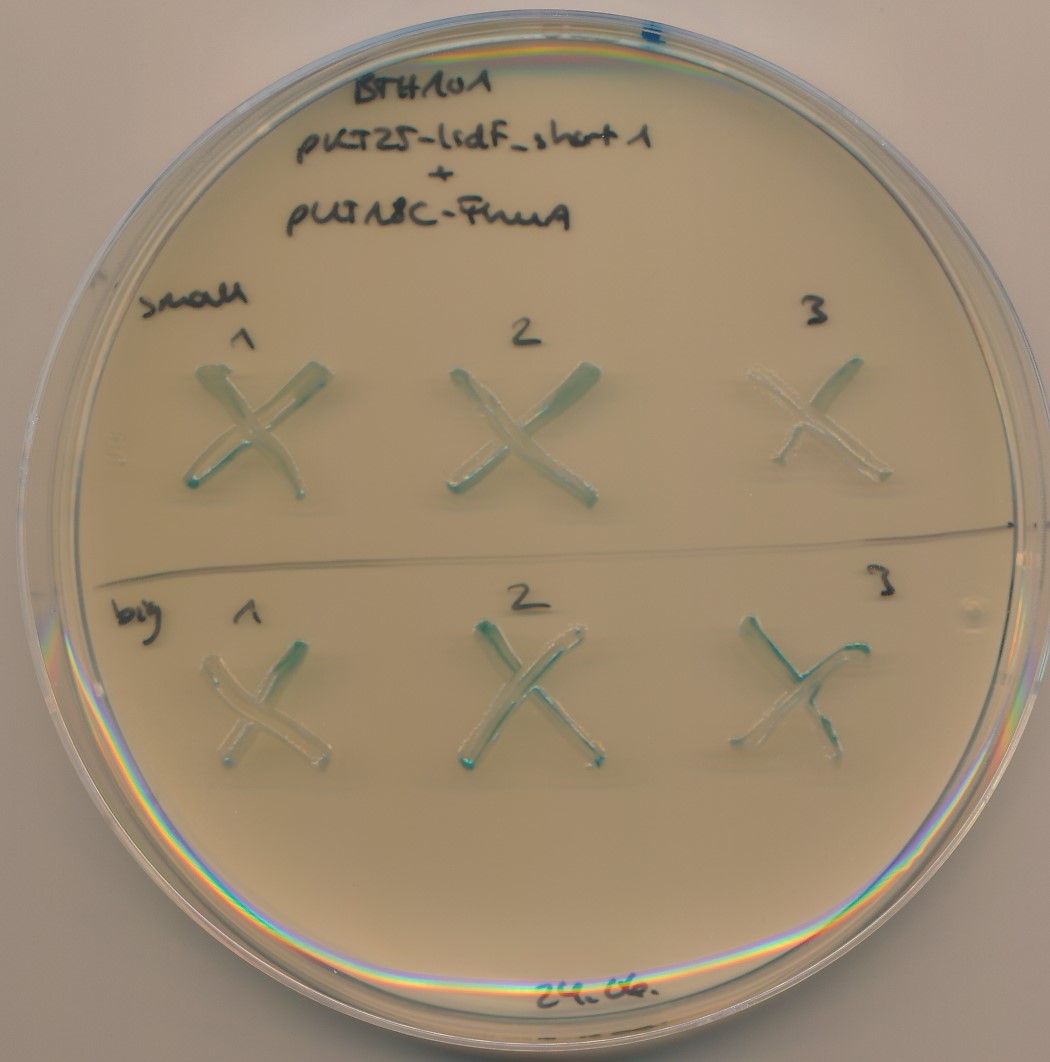

Supplement: Figure 2—source data 1. [file elife-85304-fig2-data1.zip › Figure 2e-source data/short1+FhuC_original.jpg]

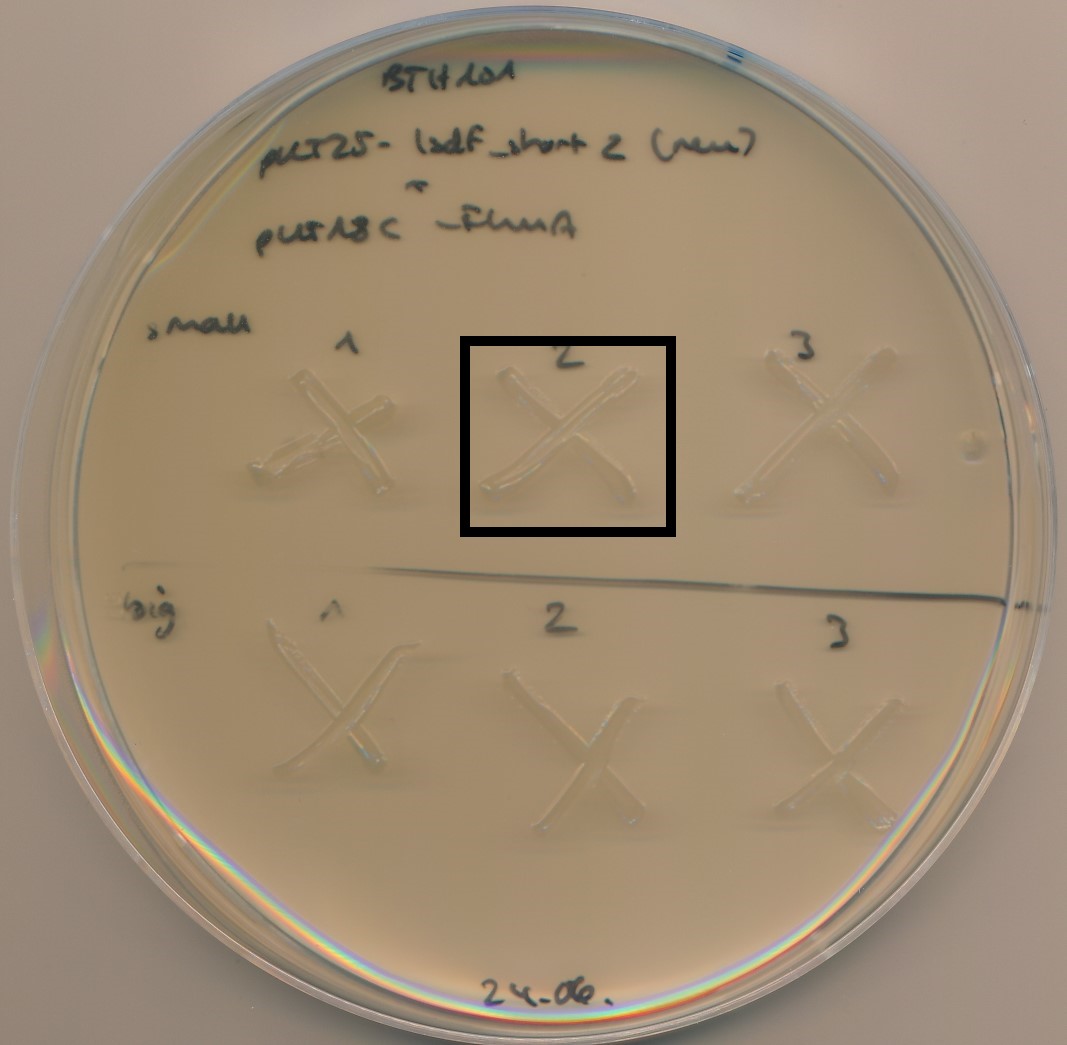

Supplement: Figure 2—source data 1. [file elife-85304-fig2-data1.zip › Figure 2e-source data/short2+FhuC_annotated.jpg]

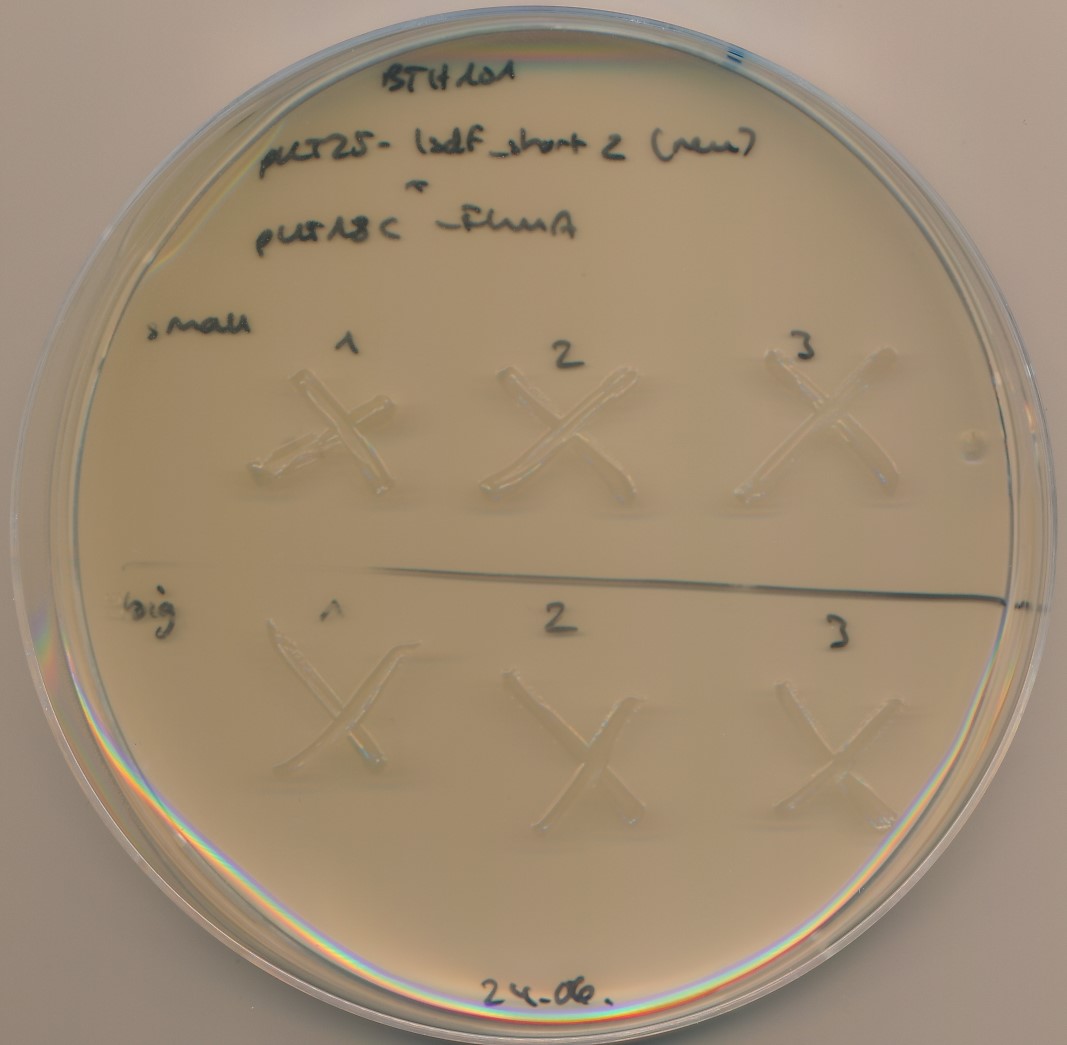

Supplement: Figure 2—source data 1. [file elife-85304-fig2-data1.zip › Figure 2e-source data/short2+FhuC_original.jpg]

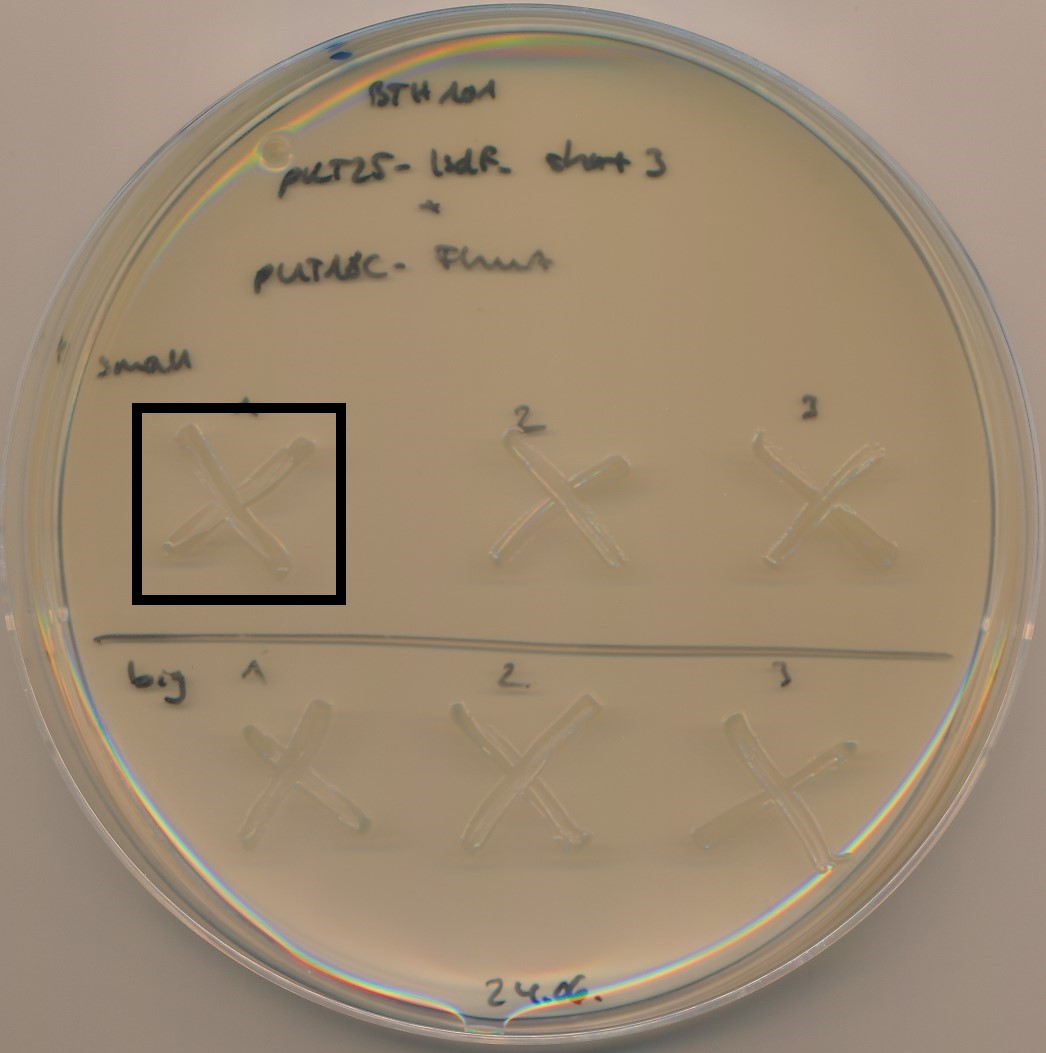

Supplement: Figure 2—source data 1. [file elife-85304-fig2-data1.zip › Figure 2e-source data/short3+FhuC_annotated.jpg]

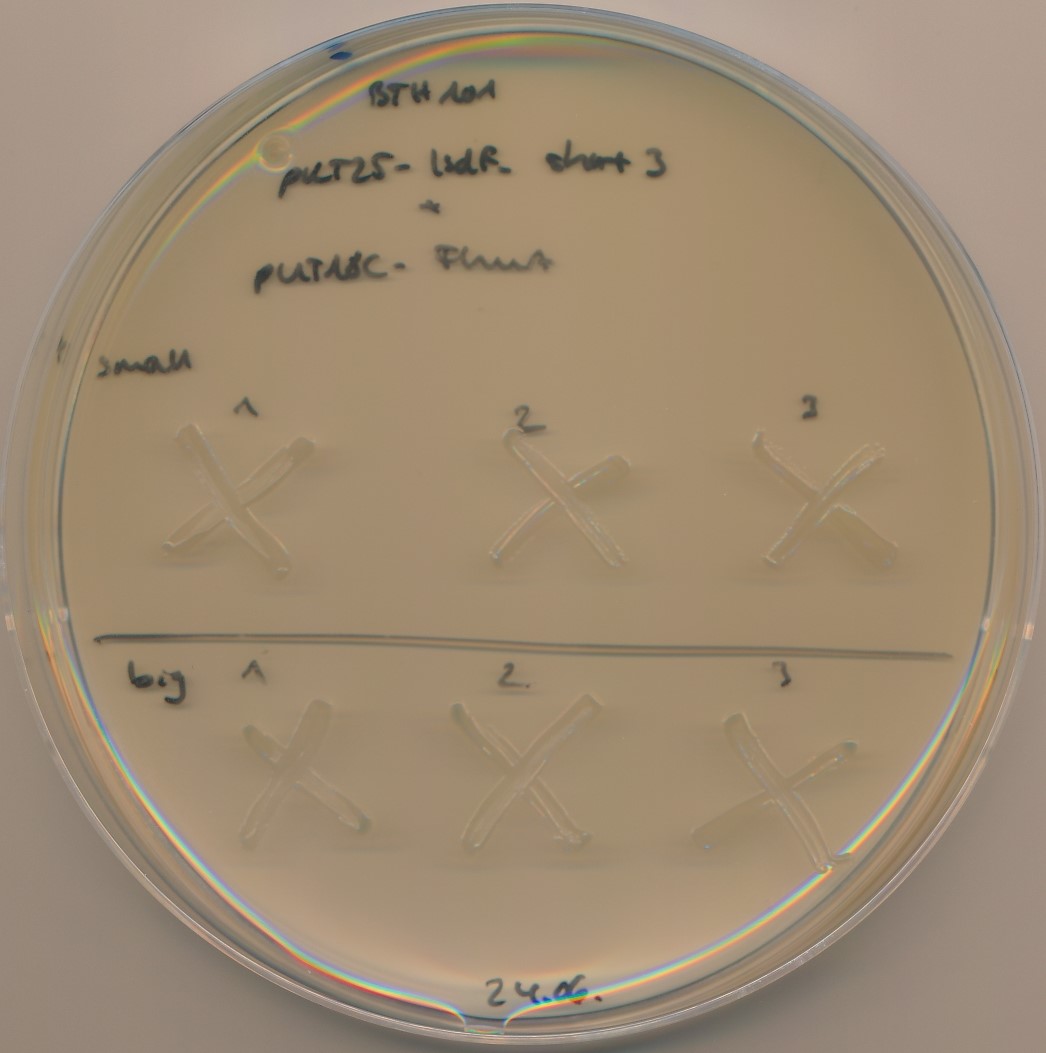

Supplement: Figure 2—source data 1. [file elife-85304-fig2-data1.zip › Figure 2e-source data/short3+FhuC_original.jpg]

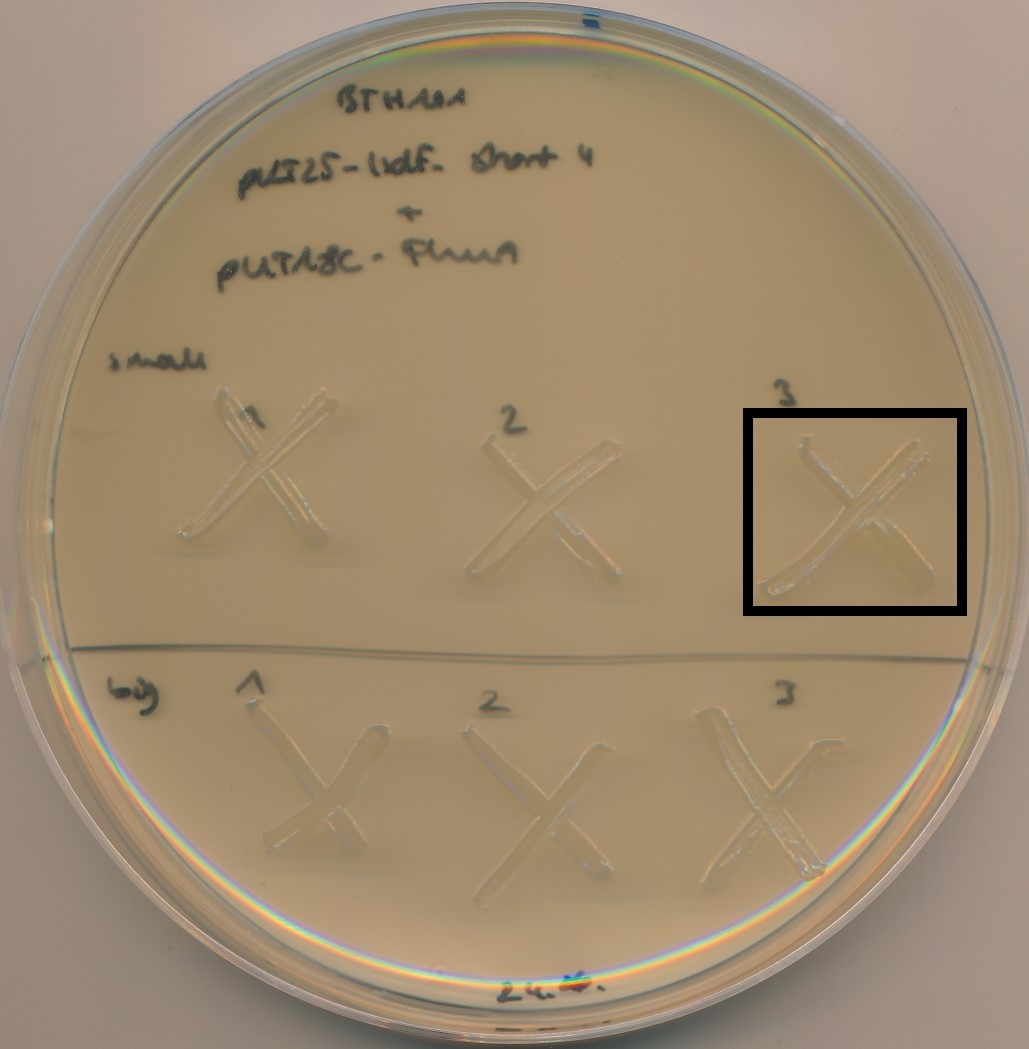

Supplement: Figure 2—source data 1. [file elife-85304-fig2-data1.zip › Figure 2e-source data/short4+FhuC_annotated.jpg]

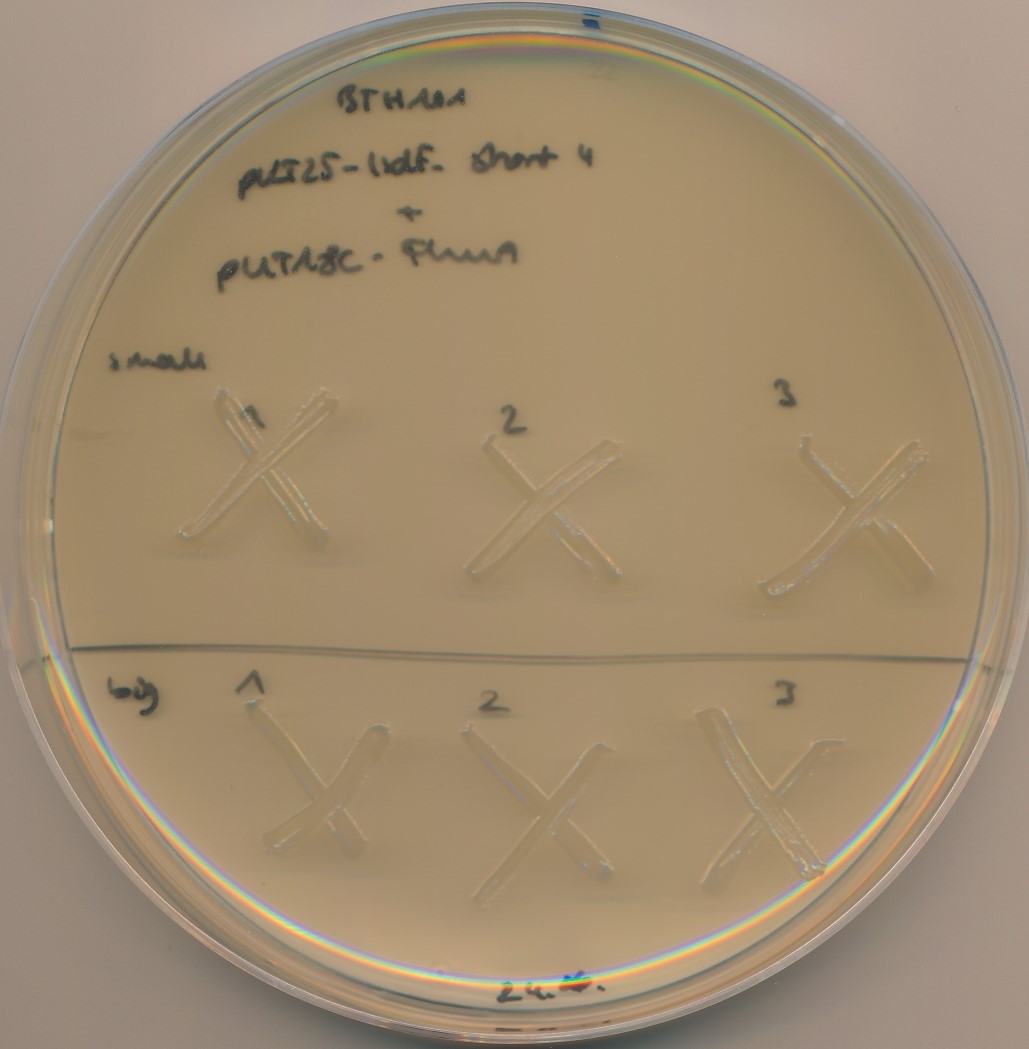

Supplement: Figure 2—source data 1. [file elife-85304-fig2-data1.zip › Figure 2e-source data/short4+FhuC_original.jpg]

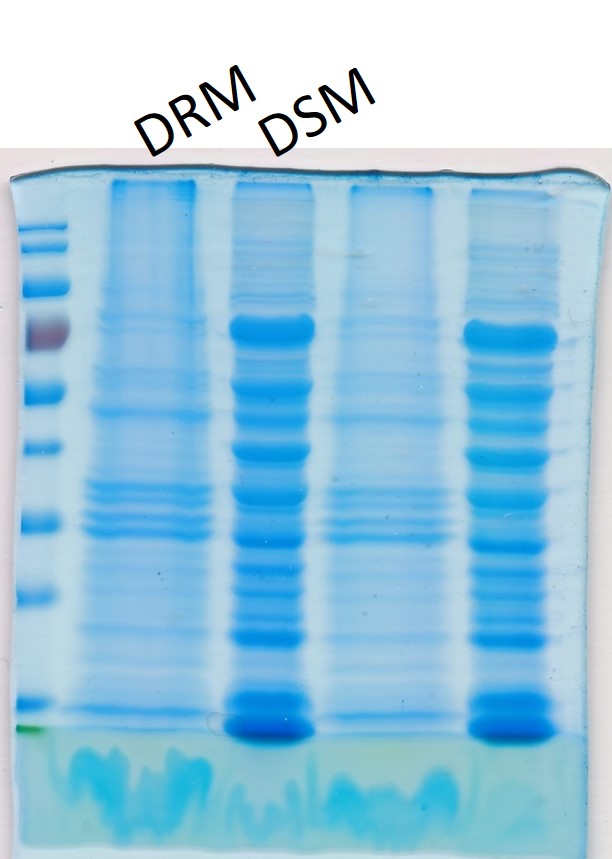

Supplement: Figure 3—source data 1. [file elife-85304-fig3-data1.zip › Figure 3a-source data_annotated.jpg]

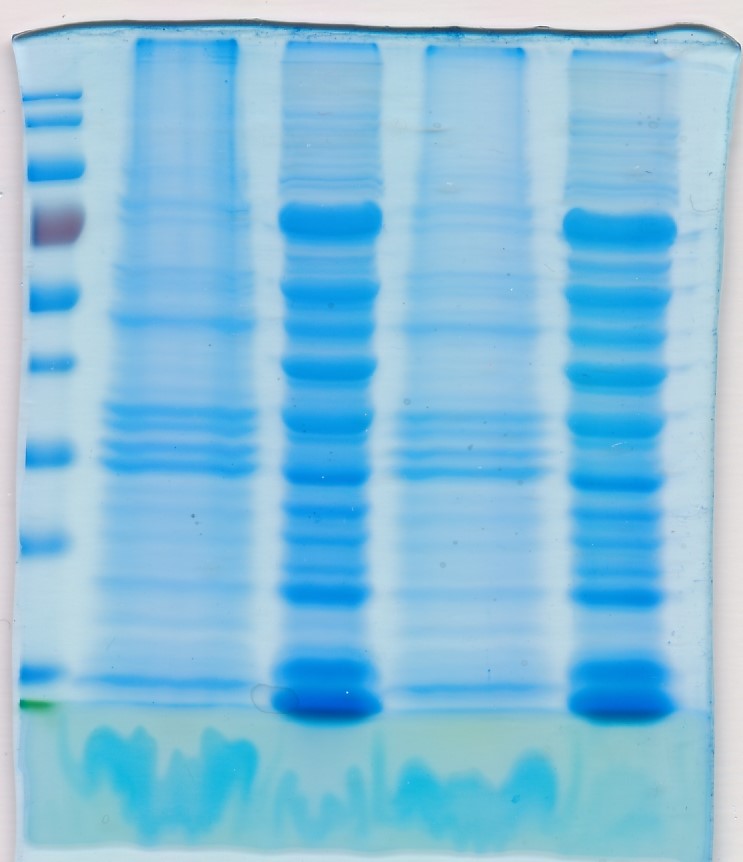

Supplement: Figure 3—source data 1. [file elife-85304-fig3-data1.zip › Figure 3a-source data_original.jpg]

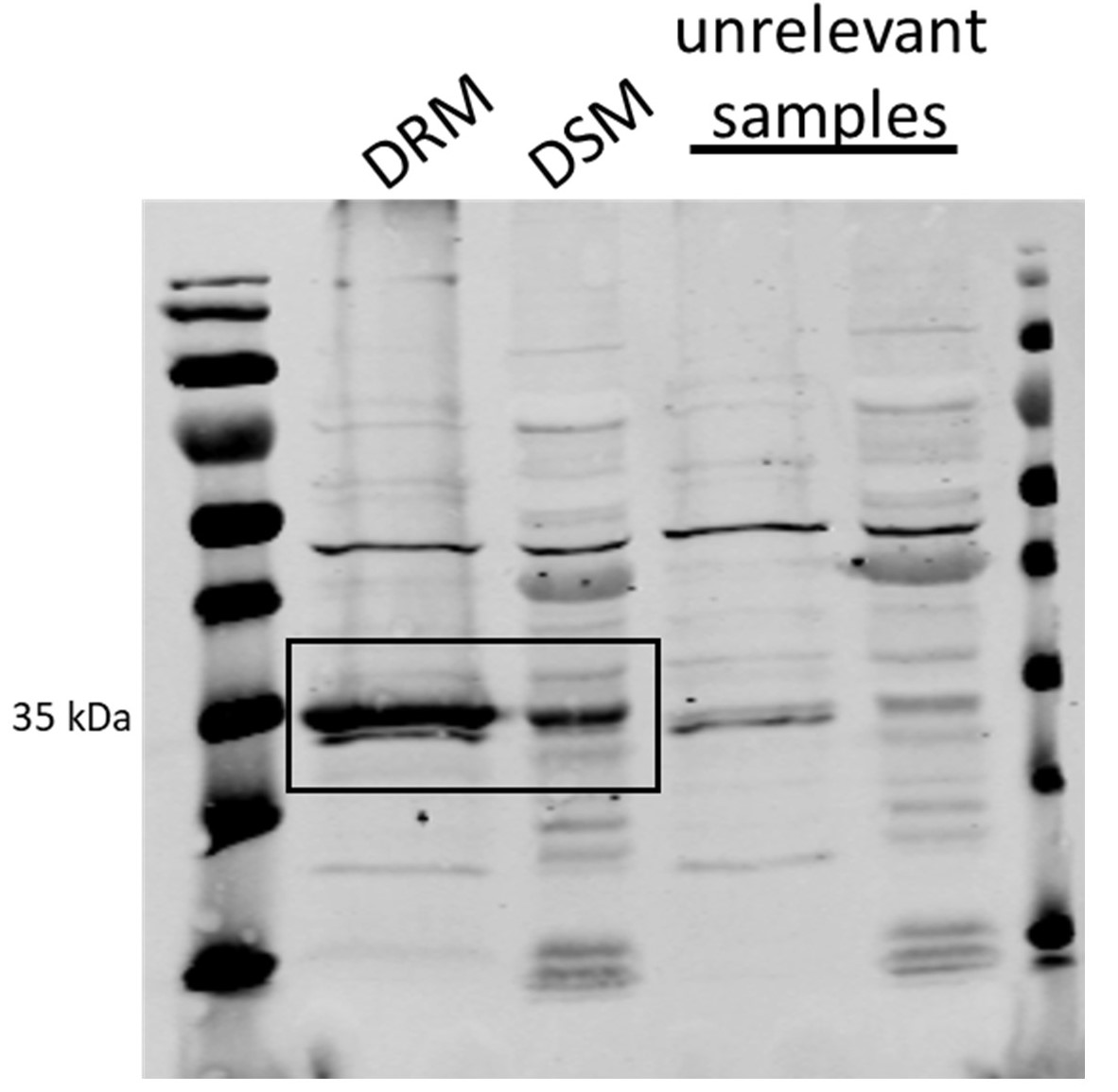

Supplement: Figure 3—source data 1. [file elife-85304-fig3-data1.zip › Figure 3b-source data_annotated.jpg]

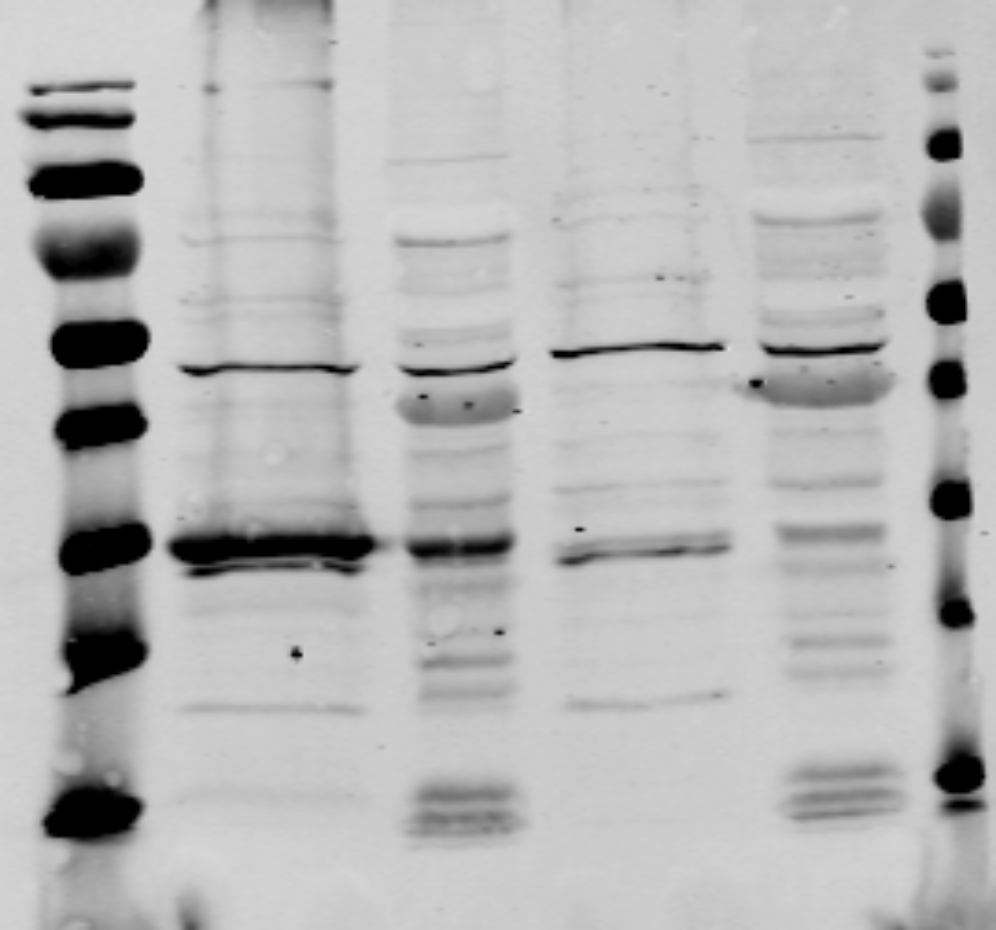

Supplement: Figure 3—source data 1. [file elife-85304-fig3-data1.zip › Figure 3b-source data_original.tif]

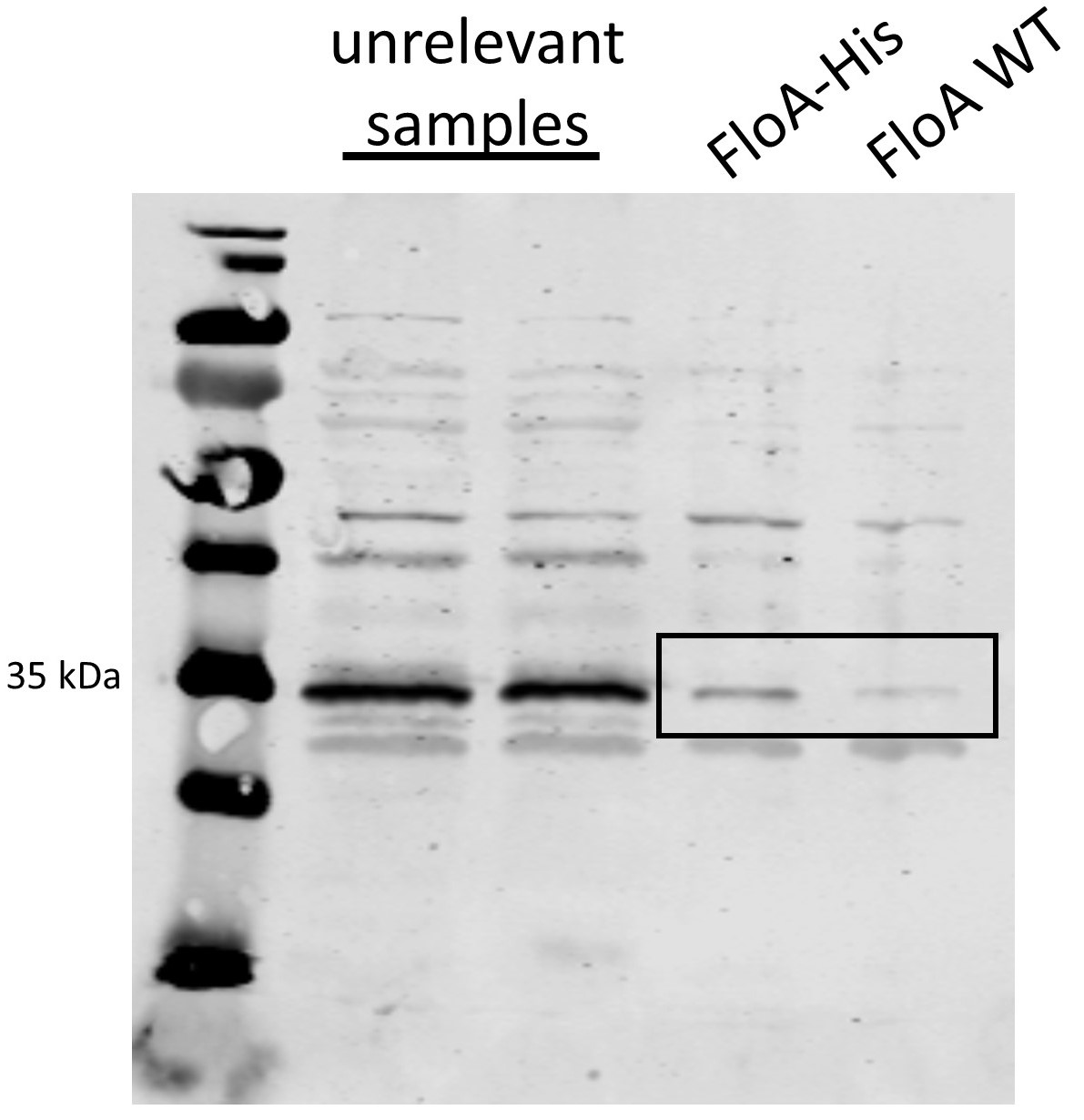

Supplement: Figure 3—source data 1. [file elife-85304-fig3-data1.zip › Figure 3c-source data_annotated.jpg]

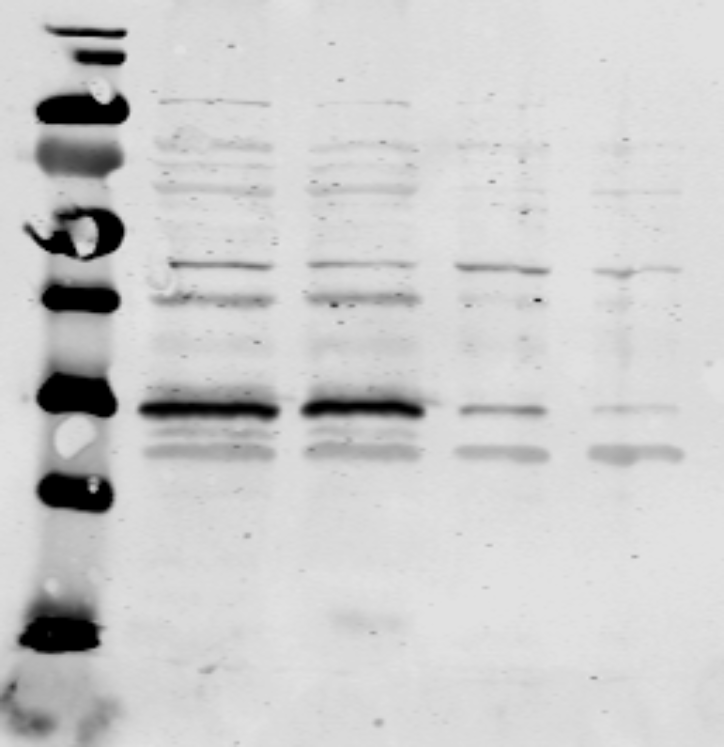

Supplement: Figure 3—source data 1. [file elife-85304-fig3-data1.zip › Figure 3c-source data_original.tif]

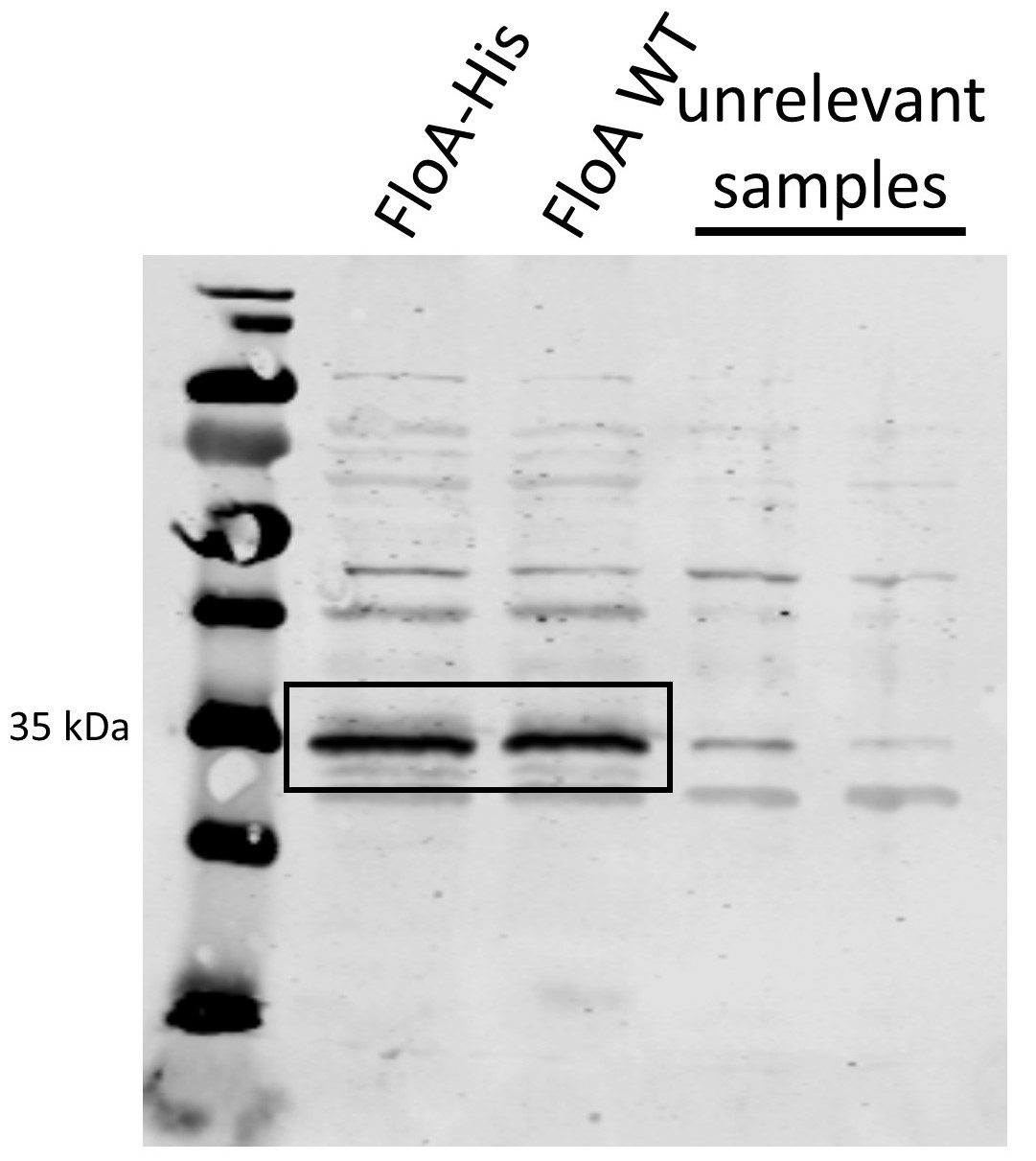

Supplement: Figure 3—figure supplement 1—source data 1. [file elife-85304-fig3-figsupp1-data1.zip › Figure 3-supplement 1_annotated.jpg]

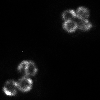

Supplement: Figure 4—source data 1. [file elife-85304-fig4-data1.zip › Figure 4-source data/4a_colocolization_example image.tif]

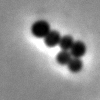

Supplement: Figure 4—source data 1. [file elife-85304-fig4-data1.zip › Figure 4-source data/4c_ floa-snap_background example image.tif]

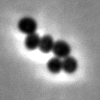

Supplement: Figure 4—source data 1. [file elife-85304-fig4-data1.zip › Figure 4-source data/4c_dfloA-background_example image.tif]

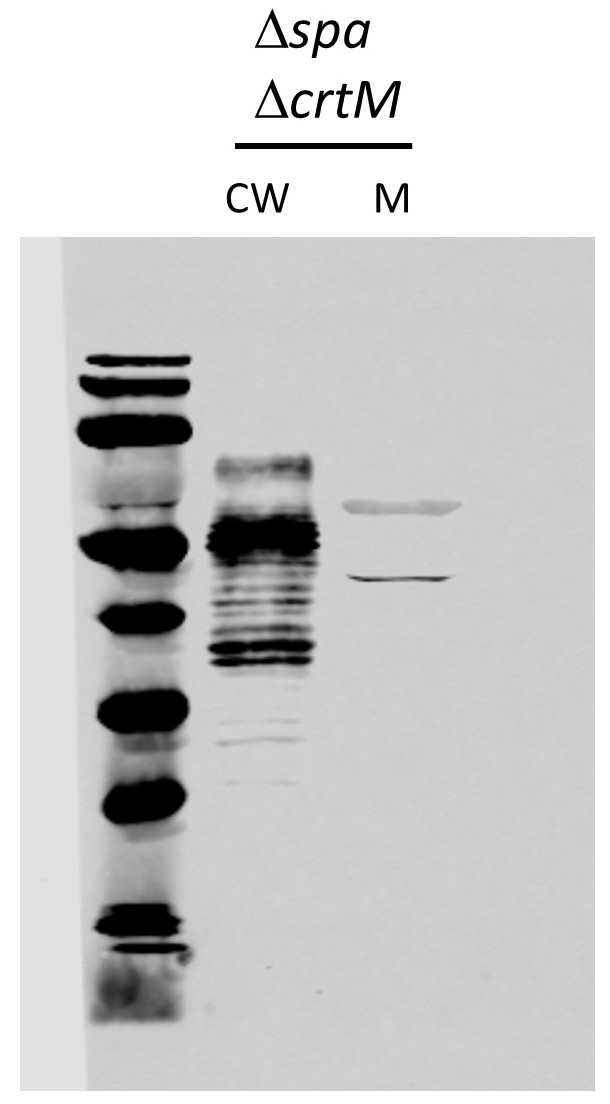

Supplement: Figure 7—source data 1. [file elife-85304-fig7-data1.zip › Figure 7-source data_annotated_2.jpg]

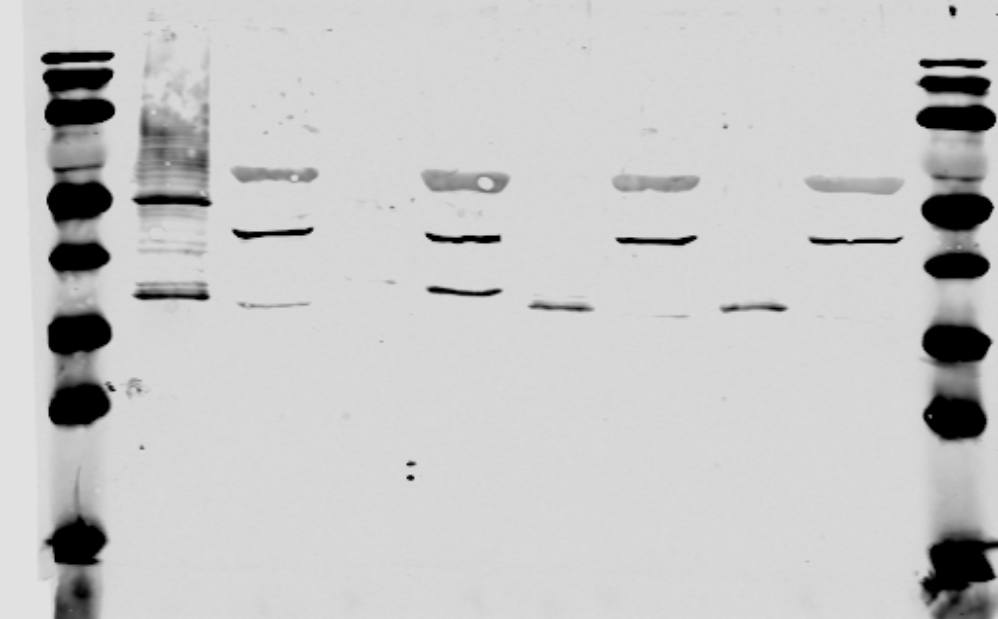

Supplement: Figure 7—source data 1. [file elife-85304-fig7-data1.zip › Figure 7-source data_original_1.tif]

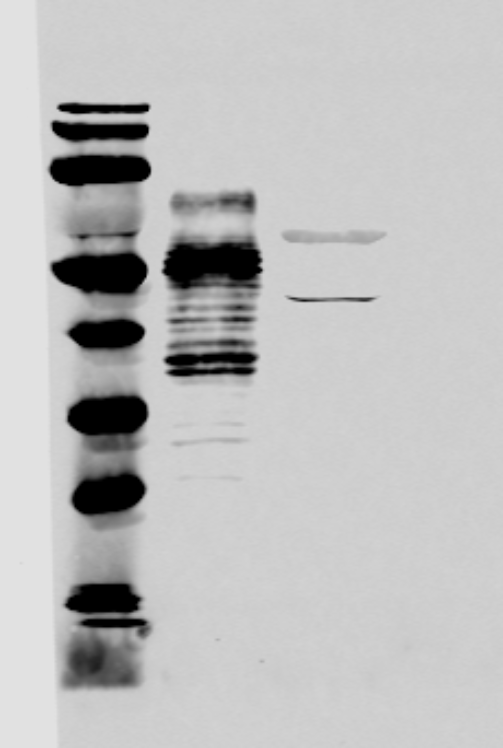

Supplement: Figure 7—source data 1. [file elife-85304-fig7-data1.zip › Figure 7-source data_original_2.tif]

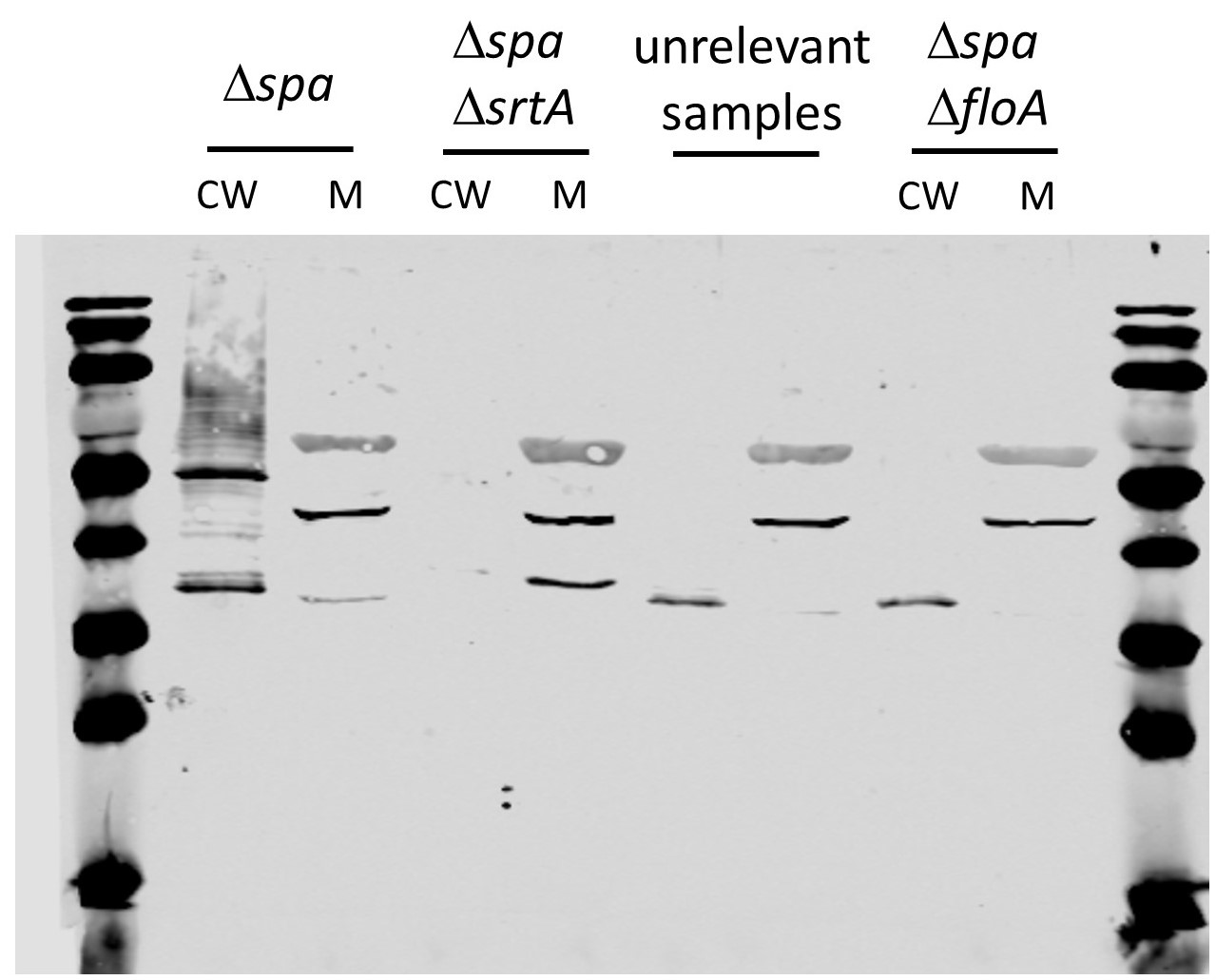

Supplement: Figure 7—source data 1. [file elife-85304-fig7-data1.zip › Figure 7_source data_annotated_1.jpg]

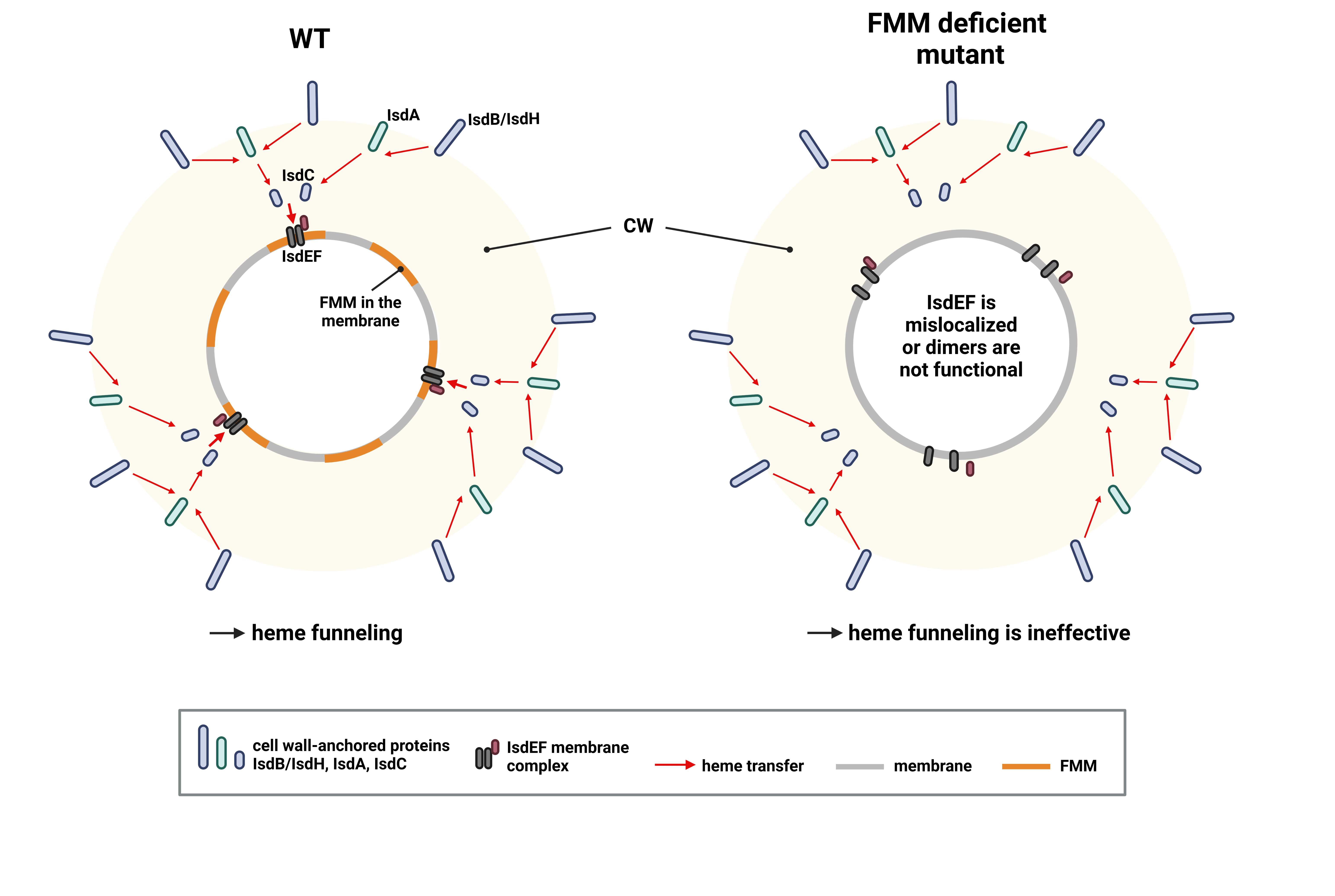

Supplement: Figure 9—source data 1. [file elife-85304-fig9-data1.zip › Figure 9.jpg]
