# Supplementary material for: Functional membrane microdomains and the hydroxamate siderophore transporter ATPase FhuC govern Isd-dependent heme acquisition in Staphylococcus aureus
Source: eLife. 2023 Apr 12;12:e85304. doi: 10.7554/eLife.85304 (PMC10147376; doi:10.7554/eLife.85304)
Supplement: Figure 2—figure supplement 1—source data 1. [file elife-85304-fig2-figsupp1-data1.zip › Figure 2-supplement 1b-source data.docx]

| Sequence and predicted topologies: (i: inside the membrane, o: outside of the membrane, M: membrane region, u: non-membrane region but location unkown)   \|  \| 1                                           41 \| \| --- \| --- \| \| Seq. \| MIKNKKKLLF LCLLVILIAT AYISFVTGTI KLSFNDLFTK FTTGSNEAVD \| \| TOPCONS \| iiiiiiiiMM MMMMMMMMMM MMMMMMMMMo oooooooooo oooooooooo \| \| OCTOPUS \| iiiiiiiiMM MMMMMMMMMM MMMMMMMMMo oooooooooo oooooooooo \| \| Philius \| iiiiiiiMMM MMMMMMMMMM MMMMMMMMMo oooooooooo oooooooooo \| \| PolyPhobius \| iiiiiiMMMM MMMMMMMMMM MMMMMMoooo oooooooooo oooooooooo \| \| SCAMPI \| iiiiiiiiiM MMMMMMMMMM MMMMMMMMMM oooooooooo oooooooooo \| \| SPOCTOPUS \| SSSSSSSSSS SSSSSSSSSS SSSooooooo oooooooooo oooooooooo \| \| [4g1uA](http://www.rcsb.org/pdb/explore/explore.do?structureId=4g1u) \| -----iiiiM MMMMMMMMMM MMMMMooooo oooooooo-- -ooooooooo \| \|  \| \| \|  \| 51                                          91 \| \| Seq. \| SIIDLRLPRI LIALMVGAML AVSGALLQAA LQNPLAEANI IGVSSGALIM \| \| TOPCONS \| oooooooMMM MMMMMMMMMM MMMMMMMMii iiiiiiiMMM MMMMMMMMMM \| \| OCTOPUS \| oooooooMMM MMMMMMMMMM MMMMMMMMii iiiiiiiMMM MMMMMMMMMM \| \| Philius \| oooooooooM MMMMMMMMMM MMMMMMMMMM MiiiiiiMMM MMMMMMMMMM \| \| PolyPhobius \| ooooooooMM MMMMMMMMMM MMMMMMMMMi iiiiiiiiMM MMMMMMMMMM \| \| SCAMPI \| ooooooMMMM MMMMMMMMMM MMMMMMMiii iiiiiiiiii iiMMMMMMMM \| \| SPOCTOPUS \| oooooooMMM MMMMMMMMMM MMMMMMMMii iiiiiiiMMM MMMMMMMMMM \| \| [4g1uA](http://www.rcsb.org/pdb/explore/explore.do?structureId=4g1u) \| oooooooooM MMMMMMMMMM MMMMMiiiii iiiiiiiiMM MMMMMMMMMM \| \|  \| \| \|  \| 101                                         141 \| \| Seq. \| RALCMLFIPQ LYFYLPLLSF IGGLIPFLII ILLHSKFRFN AVSMILVGVA \| \| TOPCONS \| MMMMMMMMoo MMMMMMMMMM MMMMMMMMMM Miiiiiiiii iMMMMMMMMM \| \| OCTOPUS \| MMMMMMMMoo MMMMMMMMMM MMMMMMMMMM Miiiiiiiii iMMMMMMMMM \| \| Philius \| MMMMMMMMoo oMMMMMMMMM MMMMMMMMMM MMMMiiiiii MMMMMMMMMM \| \| PolyPhobius \| MMMMMMMMoo oooMMMMMMM MMMMMMMMMM MMMMiiiiii iiiMMMMMMM \| \| SCAMPI \| MMMMMMMMMM MMMoMMMMMM MMMMMMMMMM MMMMMiiiMM MMMMMMMMMM \| \| SPOCTOPUS \| MMMMMMMMoo MMMMMMMMMM MMMMMMMMMM Miiiiiiiii iMMMMMMMMM \| \| [4g1uA](http://www.rcsb.org/pdb/explore/explore.do?structureId=4g1u) \| MMMMoooooo ooooMMMMMM MMMMMMMMMM Miiiiiiiii iiiiiMMMMM \| \|  \| \| \|  \| 151                                         191 \| \| Seq. \| LFVLLNGVLE ILTQNPLMKI PQGLTMKIWS DVYILAVSAL LGLILTLLLS \| \| TOPCONS \| MMMMMMMMMM MMoooooooo oooooooooo ooMMMMMMMM MMMMMMMMMM \| \| OCTOPUS \| MMMMMMMMMM MMoooooooo oooooooooo ooMMMMMMMM MMMMMMMMMM \| \| Philius \| MMMMMMMMMM MMMooooooo oooooooooo oMMMMMMMMM MMMMMMMMMM \| \| PolyPhobius \| MMMMMMMMMM MMMooooooo oooooooooo oooMMMMMMM MMMMMMMMMM \| \| SCAMPI \| MMMMMMMMMo oooooooooo oooooooooM MMMMMMMMMM MMMMMMMMMM \| \| SPOCTOPUS \| MMMMMMMMMM MMoooooooo oooooooooo ooMMMMMMMM MMMMMMMMMM \| \| [4g1uA](http://www.rcsb.org/pdb/explore/explore.do?structureId=4g1u) \| MMMMMMMMMM Mooooooooo oooooooooo oMMMMMMMMM MMMMMMMiii \| \|  \| \| \|  \| 201                                         241 \| \| Seq. \| PKLNLLNLDD IQARSIGFNI DRYRWLTGLL AVFLASATVA IVGQLAFLGI \| \| TOPCONS \| MMMiiiiiii iiiiiiiMMM MMMMMMMMMM MMMMMMMMoM MMMMMMMMMM \| \| OCTOPUS \| MMMiiiiiii iiiiiiiiii iiiMMMMMMM MMMMMMMMoM MMMMMMMMMM \| \| Philius \| MMMMMiiiii iiiiiiiiii iiiiMMMMMM MMMMMMMMMM MMMMMMMMMM \| \| PolyPhobius \| MMMMMiiiii iiiiiiiiii iiiiMMMMMM MMMMMMMMMM MMMMMMMMMM \| \| SCAMPI \| iiiiiiiiii iiiiiiiMMM MMMMMMMMMM MMMMMMMMoM MMMMMMMMMM \| \| SPOCTOPUS \| MMMiiiiiii iiiiiiiiii iiiMMMMMMM MMMMMMMMoM MMMMMMMMMM \| \| [4g1uA](http://www.rcsb.org/pdb/explore/explore.do?structureId=4g1u) \| iiiiiiiiii iiiiiiiiii iiiiiMMMMM MMMMMMMMMM MooMMMMMMM \| \|  \| \| \|  \| 251                                         291 \| \| Seq. \| IVPHVVRKLV GGNYRVLIPF STVIGAWLLL VADLLGRVIQ PPLEIPANAI \| \| TOPCONS \| MMMMMMMMMM iiiiiMMMMM MMMMMMMMMM MMMMMMoooo ooMMMMMMMM \| \| OCTOPUS \| MMMMiiiiii iiiiMMMMMM MMMMMMMMMM MMMMMooooo oMMMMMMMMM \| \| Philius \| Mooooooooo oooooMMMMM MMMMMMMMMM MMMMMMiiii iiiiMMMMMM \| \| PolyPhobius \| MMMooooooo oooooMMMMM MMMMMMMMMM MMMMMMiiii iiiMMMMMMM \| \| SCAMPI \| MMMMMMMMMM iiiiiMMMMM MMMMMMMMMM MMMMMMoooo ooMMMMMMMM \| \| SPOCTOPUS \| MMMMiiiiii iiiiMMMMMM MMMMMMMMMM MMMMMooooo oMMMMMMMMM \| \| [4g1uA](http://www.rcsb.org/pdb/explore/explore.do?structureId=4g1u) \| MMMMMMMMMi iiiiiiiiiM MMMMMMMMMM MMMMMooooo ooooooMMMM \| \|  \| \|  \|  \| 301                   321 \| \| --- \| --- \| \| Seq. \| LMIVGGPMLI YLICQSQRNR I \| \| TOPCONS \| MMMMMMMMMM MMMiiiiiii i \| \| OCTOPUS \| MMMMMMMMMM MMiiiiiiii i \| \| Philius \| MMMMMMMMMM MMMMoooooo o \| \| PolyPhobius \| MMMMMMMMMM MMMMoooooo o \| \| SCAMPI \| MMMMMMMMMM MMMiiiiiii i \| \| SPOCTOPUS \| MMMMMMMMMM MMiiiiiiii i \| \| [4g1uA](http://www.rcsb.org/pdb/explore/explore.do?structureId=4g1u) \| MMMMMMMMMM MMiiiiiiii - \| |
| --- | --- | --- | --- | --- | --- | --- | --- | --- | --- | --- | --- | --- | --- | --- | --- | --- | --- | --- | --- | --- | --- | --- | --- | --- | --- | --- | --- | --- | --- | --- | --- | --- | --- | --- | --- | --- | --- | --- | --- | --- | --- | --- | --- | --- | --- | --- | --- | --- | --- | --- | --- | --- | --- | --- | --- | --- | --- | --- | --- | --- | --- | --- | --- | --- | --- | --- | --- | --- | --- | --- | --- | --- | --- | --- | --- | --- | --- | --- | --- | --- | --- | --- | --- | --- | --- | --- | --- | --- | --- | --- | --- | --- | --- | --- | --- | --- | --- | --- | --- | --- | --- | --- | --- | --- | --- | --- | --- | --- | --- | --- | --- | --- | --- | --- | --- | --- | --- | --- | --- | --- | --- | --- | --- | --- | --- | --- | --- | --- | --- | --- | --- | --- | --- | --- | --- | --- | --- | --- |

|  |
| --- |
