# Supplementary material for: Functional membrane microdomains and the hydroxamate siderophore transporter ATPase FhuC govern Isd-dependent heme acquisition in Staphylococcus aureus
Source: eLife. 2023 Apr 12;12:e85304. doi: 10.7554/eLife.85304 (PMC10147376; doi:10.7554/eLife.85304)
Supplement: Figure 2—figure supplement 1—source data 1. [file elife-85304-fig2-figsupp1-data1.zip › Figure 2-supplement 1c-source data.docx]

CLUSTAL O(1.2.4) multiple sequence alignment

MntB --RPLMISTFDPTFSRMSGLN-------- 19

SirC -IPKMKISILDDPVAIGLGLHVQRMK--- 25

IsdF -----NLLNLDDIQARSIG---------- 14

FhuG KSNTLNIIHTGDNIARGLGVRLSRERLIL 29

SirB ----INILMTSDDIATGLG---------- 15

HtsC ---QLDVLNLGDAVATALGLKVKTIK--- 23

FhuB ----LTILNLGESLAKGLGQ--------- 16

HtsB ------IMELGDDIAKGLGQNINKVR--- 20

: . : *
